# Genome-Wide Identification and Expression Analysis of the *WRKY* Gene Families in *Vaccinium bracteatum*

**DOI:** 10.3390/ijms26167835

**Published:** 2025-08-13

**Authors:** Haijing Du, Jianqiang Zhou, Xiaoran Liang, Yufei Chen, Xiaohui Liu, Cheng Zhen, Hong Zhang, Jiaxin Xiao, Xuan Gao

**Affiliations:** 1 Anhui Provincial Key Laboratory of Molecular Enzymology and Mechanism of Major Metabolic Diseases, College of Life Sciences, Anhui Normal University, Wuhu 241000, China; 18355632985@163.com (H.D.); cyf2130035271@163.com (Y.C.); liuxiaohui@ahnu.edu.cn (X.L.); 2 Engineering Research Center of Biofilm Water Purification and Utilization Technology of Ministry of Education, Anhui University of Technology, Ma’anshan 243032, China; 3 Anhui Provincial Engineering Research Centre for Molecular Detection and Diagnostics, College of Life Sciences, Anhui Normal University, Wuhu 241000, China; 22111703025@ahnu.edu.cn (J.Z.); liangxiaoran@ahnu.edu.cn (X.L.); c2320288389@163.com (C.Z.); 18672312335@163.com (H.Z.)

**Keywords:** *Vaccinium bracteatum*, *WRKY* transcription factor, genome-wide analysis, chromosome localization, bioinformatic, abiotic stress, expression profiling

## Abstract

The *WRKY* gene family is a widely distributed and highly conserved transcription factor (TF) family in plants, with its members playing key roles in plant growth and development, stress response, and metabolism. Although *WRKY* TFs have been extensively studied in many plant species, research on the *WRKY* gene family in *Vaccinium bracteatum* Thunb. remains limited. Therefore, integrating molecular biology and bioinformatics approaches to further explore the *WRKY* gene family in *V. bracteatum* is of considerable scientific importance. In this study, we employed various online tools to obtain genomic and expression data, which were subsequently analyzed to determine the composition, evolutionary relationships, and functions of *WRKY* family genes in *V. bracteatum*. A total of 66 *WRKY* genes (*VaWRKY*) were identified, named based on homology alignment. Phylogenetic analysis classified the 66 *VaWRKYs* into three major clades and seven subclades. Sequence and structural analyses of *VaWRKY* genes provided insights into their evolutionary and functional characteristics. Expression profile analysis revealed significant differences in the expression of 12 *VaWRKY* genes at various stages of fruit development. Protein interaction analysis further indicated that *VaWRKY* genes are functionally diverse, playing important roles in stress response, seed germination regulation, and plant growth and development. In summary, we have a deeper understanding of *VaWRKY* genes, and systematic analysis of structure, evolutionary characteristics, and expression patterns plays an important role in analyzing its biological functions, molecular breeding, and enhancing economic value.

## 1. Introduction

*Vaccinium bracteatum* Thunb., commonly known as the Chinese wild blueberry, is an evergreen shrub or small tree belonging to the Ericaceae family. It is one of the most widely distributed species of the *Vaccinium* genus in China, known for its significant health benefits, medicinal properties, and ornamental value, making it an important subject for research. Despite its potential, the interest in *V. bracteatum* has been relatively limited due to its poor palatability, which has resulted in a smaller research community focused on this species. *V. bracteatum* is closely related to the commercially popular blueberry, and as a native species, it demonstrates a remarkable resilience to environmental stressors. This plant is rich in essential nutrients and bioactive compounds, including anthocyanins and flavonoids, contributing to its pharmacological profiles and potential health benefits. Understanding the adaptive mechanisms of *V. bracteatum* in response to abiotic and biotic stresses not only adds to the knowledge of plant resilience but also offers potential applications in agronomy and horticulture.

The *WRKY* transcription factors play critical roles in various biological processes in plants, including growth [1], development [2,3,4], and stress responses [5]. These factors are primarily known for their involvement in regulating gene expression associated with plant defense mechanisms [6,7,8], hormone signaling [9,10], and abiotic stress tolerance [11]. Recent studies have expanded their functional relevance beyond stress responses, highlighting their participation in processes such as flowering [12], leaf senescence [13], and, particularly, fruit development [14]. This emphasizes the multifaceted roles of WRKY proteins in plant biology.

Research on *WRKY* transcription factors has been carried out across various plant species. Among these, studies have been notably focused on economically important crops like soybean (*Glycine max*), *Arabidopsis thaliana*, rice (*Oryza sativa*), and citrus species. Additionally, recent genomic investigations have identified *WRKY* factors in a diverse range of plants, including the model organism tobacco (*Nicotiana tabacum*), barley (*Hordeum vulgare*), and various ornamental and fruit-bearing species like apple (*Malus domestica*). This broad research scope underscores the significance of *WRKY* genes in understanding plant development, particularly in the context of fruit maturation and quality.

Focusing specifically on fruit development, several *WRKY* transcription factors have been implicated in this process. Noteworthy examples include *MaWRKY49* in banana (*Musa acuminata*) [14], which regulates pectate lyase genes during fruit ripening, and *GmWRKY164* in soybean [15], which enhances resistance to viral infections affecting fruit production. Experimental methods such as gene expression analysis combined with knockout and overexpression studies have provided insights into their roles. Additionally, techniques like CRISPR/Cas9 gene editing and loss-of-function assays have further validated the functional contributions of specific *WRKY* factors in promoting fruit development and ripening [16].

Investigating the growth development and stress regulation mechanisms in *V. bracteatum* is invaluable, yet the underlying mechanisms remain poorly understood. An essential aspect of this research involves *WRKY* transcription factors, which play a crucial role in plant adaptive responses and developmental processes. Further exploration into the *WRKY* family within *V. bracteatum* will enhance our comprehension of its unique traits and resilience, paving the way for future studies aimed at harnessing its potential in both medicinal and ecological contexts.

In this study, we conducted a comprehensive analysis of the *WRKY* transcription factor family in *V. bracteatum* using genomic sequencing data, identifying a total of 66 *WRKY* genes. We performed phylogenetic analyses to understand the evolutionary relationships among these genes and conducted gene structure and domain analyses to characterize their functional attributes. Additionally, chromosomal localization and synteny analyses were employed to further elucidate the genomic context of these *WRKY* genes in *V. bracteatum*. Building on these foundational analyses, we carried out transcriptome sequencing of fruit at various maturity stages to identify which *WRKY* genes are modulated during fruit development. This stage of our research is critical, as it allows us to pinpoint the regulatory roles of *WRKY* transcription factors in the maturation process of the fruit. Furthermore, we performed protein-protein interaction and 3D structural analyses of these transcription factors, enabling us to predict their functional networks and interactions within the plant system.

Through these multifaceted analyses, our study aims to reveal the evolutionary significance of *WRKY* transcription factors in *V. bracteatum* and to clarify their potential regulatory roles during fruit maturation. Understanding these mechanisms will provide valuable insights into the adaptive strategies of this species and may inform future studies in plant biochemistry and agriculture, particularly for crops that share similar stress resilience traits.

## 2. Results

### 2.1. Identification of WRKY Families in V. bracteatum

Using the FASTA-formatted file of *VaWRKY* protein sequences (“Va” is used to name *WRKY* genes in this article, and the recommended standard name is “VbG1”), HMM analysis was performed, using the BLASTp tool in the NCBI database for comparison. A total of 66 valid *VaWRKY* family members were identified in the *V. bracteatum* genome, named *VaWRKY2-1* to *VaWRKYSUSIBA2* (Table S1), and classified into three categories based on their phylogenetic branches. Subsequently, the protein molecular weight, isoelectric point, amino acid length, total number of negatively charged residues, and subcellular localization of candidate *VaWRKY* family members were predicted using ExPASy and Plant-mPLoc online websites. The encoded amino acid length ranges from 75 to 820 aa, with an average length of 376.06 aa; the molecular weight varies from 8564.78 to 90,316.86 Da, with an average of 41,592.42 Da; the theoretical isoelectric point ranges from 4.54 to 10.14; the total number of negatively charged residues ranges from 8 to 94, and the total number of positively charged residues ranges from 17 to 91; the instability index ranges from 35.73 to 75.32, mostly indicating unstable proteins; the aliphatic amino acid index ranges from 41.34 to 99.14, all being lipophilic proteins; the average hydrophobicity ranges from −1.184 to −0.223, all being hydrophilic proteins. This indicates that the physicochemical properties of proteins expressed by different *VaWRKY* genes vary significantly and may play unique roles in various microenvironments. Most *VaWRKY* proteins are distributed in the cell nucleus, possibly involved in gene expression regulation and signal transduction; additionally, *VaWRKY50-1* protein is still present in the cell membrane, suggesting its role in substance transport and cell recognition adhesion, while *VaWRKY33-1* protein is also found in chloroplasts, reflecting the diversity of *VaWRKY* gene functions.

### 2.2. Phylogenetic Analysis of WRKY Gene Families in V. bracteatum

**Figure 1:**
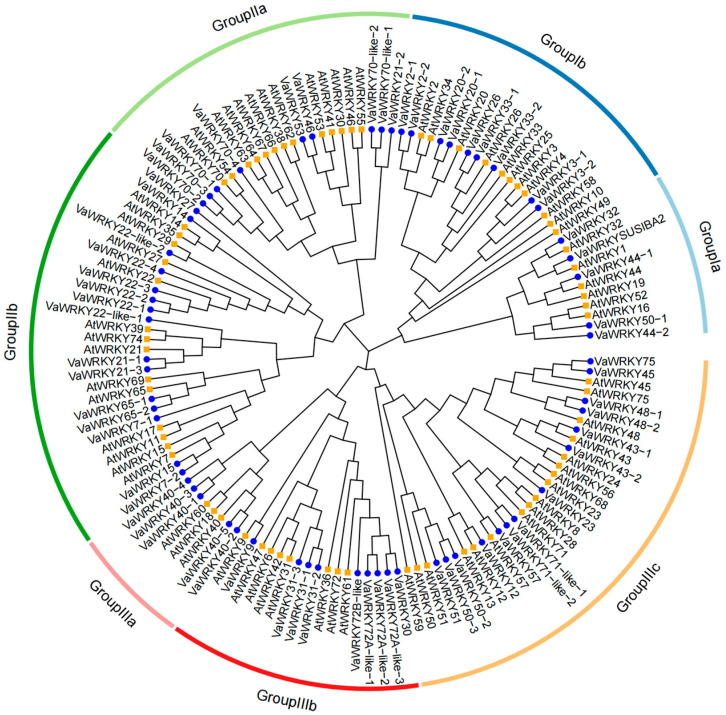
The phylogenetic relationship between *VaWRKY* and *AtWRKY*.

To explore the evolutionary relationships of the *WRKY* genes in the *V. bracteatum* family, 66 *VaWRKY* genes and 72 *AtWRKY* genes were selected for phylogenetic analysis. The phylogenetic tree was constructed using the NJ method through ClustalX2.1 software (bootstrap value of 1000), showing the phylogenetic relationship between *WRKY* genes of *V. bracteatum* and *WRKY* genes of *Arabidopsis thaliana*. According to the *AtWRKY* classification scheme and the comparison of *VaWRKY* and *AtWRKY* genes, the genes were divided into three main groups and seven subgroups, which were represented by different colors (Figure 1). The 66 *VaWRKY* genes were divided into three major groups (Groups I, II, and III) according to the branches in phylogenetic tree. The largest group is Group III, containing 28 *VaWRKY* genes, followed by Group II (24 proteins) and Group I (14 proteins). In addition, the three major groups were further divided into seven subgroups, including Ia (5), Ib (9), IIa (9), IIb (15), IIIa (5), IIIb (9), and IIIc (14). Based on the figure, the scattered distribution of *VaWRKY* in each subgroup indicated that amplification of the *WRKY* family occurred before the differentiation of *P. grandiflorus* and *A. thaliana* [17].

To elucidate the distinctive characteristics of the *WRKY* domain in each *VaWRKY* protein, the sequences of *VaWRKY* proteins were used in multiple sequence alignment analysis [18]. To characterize the conserved features of *WRKY* domains in *VaWRKY* proteins, we conducted multiple sequence alignment focusing on the core 60-amino-acid region comprising this functional motif. The *WRKY* transcription factor family is characterized by its highly conserved WRKYGQK heptapeptide domain. However, the mutations of the domain were identified in some genes. For example, in *VaWRKY50*, *VaWRKY50-3,* and *VaWRKY50-2*, “WRKYGQK” was substituted as “WRKYGKK,” and in *VaWRKY26* and *VaWRKY70-like-1*, it was “WRKYGEK.” Especially in *VaWRKY27*, which contains “WRKYGRK,” this mutation was founded in the only gene. Group I contain 14 members, and most of them contain two *WRKY* domains.

### 2.3. Gene Structure and Conserved Domain Analysis of WRKY Gene Families in V. bracteatum

**Figure 2:**
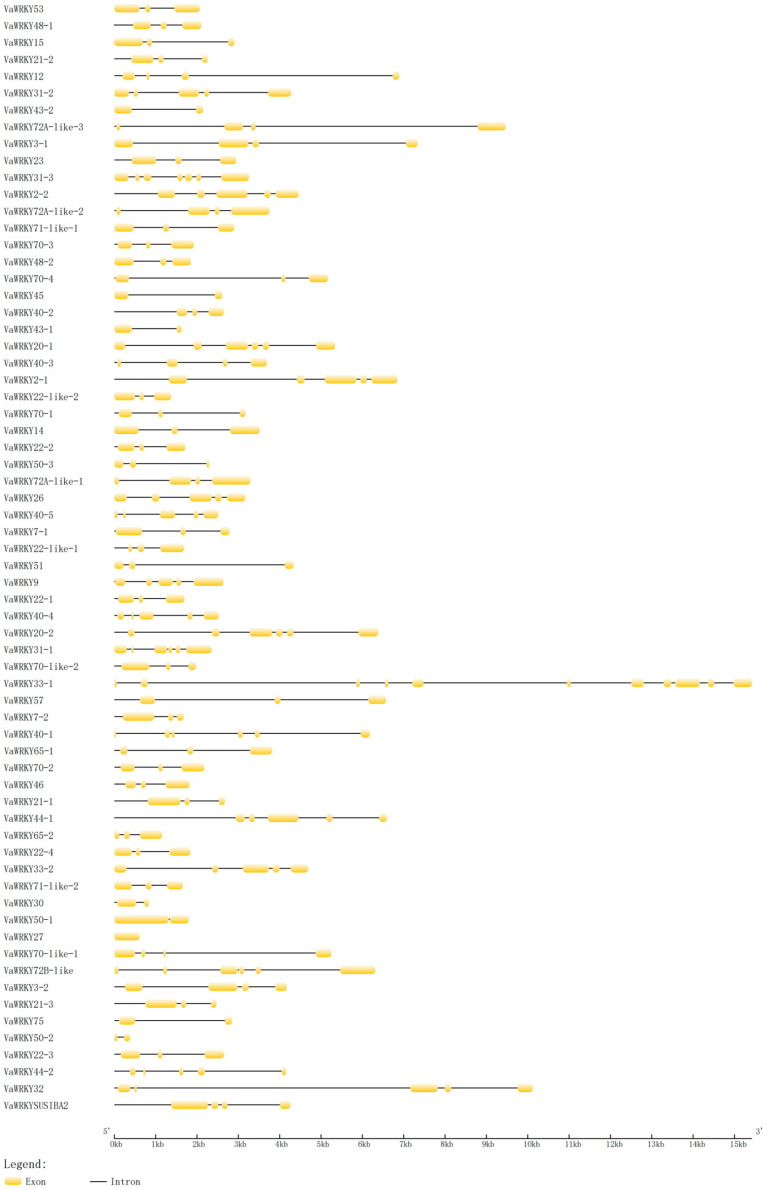
Analysis of gene structure of *VaWRKY* gene family.

To correctly understand the structural evolution of the *VaWRKY* gene, we constructed a structural map based on the genome sequence of *V. bracteatum*, analyzing the positions of exons and introns within the gene. The figure shows the exon and intron structure of genes in the *VaWRKY* gene family. Exons and introns are represented by yellow boxes and black lines, respectively. The scale at the bottom measures the length of exons and introns (Figure 2). Among the 66 genes screened, the number of exons varied from 1 to 11, and the number of introns ranged from 0 to 10. The number of exons and introns differed among genes: 31 (46.97%) genes contained three exons, nine (13.64%) genes contained four exons, and 11 (16.67%) genes contained five exons. Most genes had between three and five exons, similar to *A. thaliana*, which is related to the compactness and functional conservation of the genes. *VaWRKY33-1* had the most exons (11) and introns (10), highlighting the complexity of gene structure. Moreover, the exon/intron length ratio for most genes was less than 1, but in a few cases, such as *VaWRKY53*, *VaWRKY22-like-1*, *VaWRKY22-like-2*, *VaWRKY30*, and *VaWRKY50-1*, the length of exons was significantly greater than that of introns, suggesting specific evolutionary paths for these genes. Notably, *VaWRKY27* contained only one exon without any introns, indicating that the *VaWRKY* gene may have undergone events of exon gain or intron loss during its evolution.

**Figure 3:**
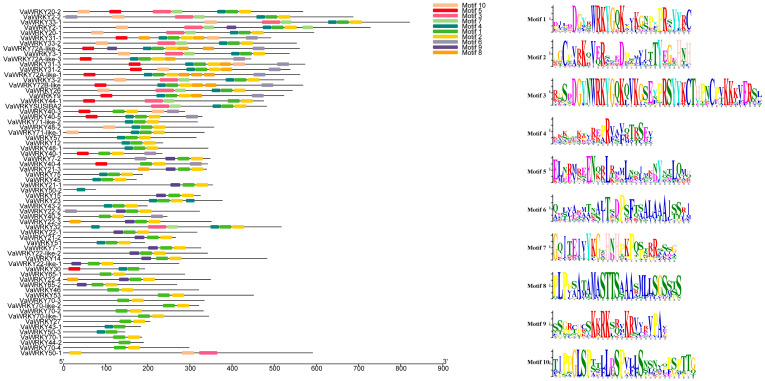
Conserved motifs of the *VaWRKY* gene family. The ten expected motifs are represented by colored boxes, and the theme logo is below.

To study the structural diversity of *WRKY* transcription factors in the *V. bracteatum*, we used the MEME5.5.5 online tool to analyze conserved motifs in *WRKY* proteins, predicting a total of 10 conserved motifs (Motif 1 to Motif 10) (Figure 3). The sequences are shown in the figure, where motif1 and motif3 contain the *WRKY* heptapeptide structure, and motif2 contains a zinc finger domain. The results indicate that different types of *WRKY* genes in the *V. bracteatum* have varying numbers and types of motifs. Most *WRKY* proteins contain motif1, suggesting that motif1 is highly conserved among all *WRKY* proteins in the *V. bracteatum*. Only the genes *VaWRKY50-1* and *VaWRKY50-2* do not contain it, with *VaWRKY50-1* containing both motif3 and a zinc finger domain, which may indicate that they are zinc-dependent transcription factors. *VaWRKY50-2*, however, contains only motif4, whose function remains unclear, possibly due to functional redundancy. Moreover, in most *VaWRKY* proteins, the conserved motifs motif1 and motif2 always appear in pairs, with the sequence consistently being motif1 and motif2, indicating that these two motifs work together to perform functions in biological processes.

### 2.4. Domain Analysis of the VaWRKY Gene Families

**Figure 4:**
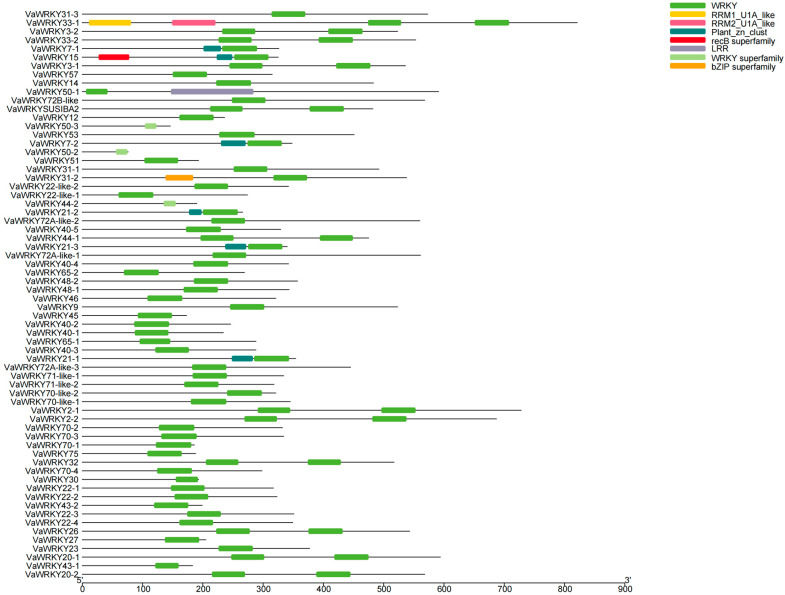
Domain analysis of the *VaWRKY* gene family. Different colors represent different domains.

In the analysis of 66 *VaWRKY* genes encoding proteins, 12 proteins contain two *WRKY* domains, while the rest have only one *WRKY* domain (Figure 4). Most proteins contain only one *WRKY* conserved domain. The N-terminal of the *WRKY* domain in *VaWRKY50-1* is incomplete, and the C-terminal of the *WRKY* domains in *VaWRKY50-2*, *VaWRKY50-3*, *VaWRKY44-2*, and *VaWRKY30* are incomplete. *VaWRKY7-1*, *VaWRKY7-2*, *VaWRKY21-1*, *VaWRKY21-2*, *VaWRKY21-3*, and *VaWRKY15* all have a closely linked plant zinc cluster domain before their *WRKY* domains. Additionally, *VaWRKY50-1* has a leucine-rich repeat sequence. *VaWRKY33-1* contains two RNA recognition motifs (RRM1_U1A-like and RRM2_U1A-like), suggesting that the protein may function with RNA involvement.

### 2.5. Chromosome Localization and Collinearity Analysis of the VaWRKY Gene Families

**Figure 5:**
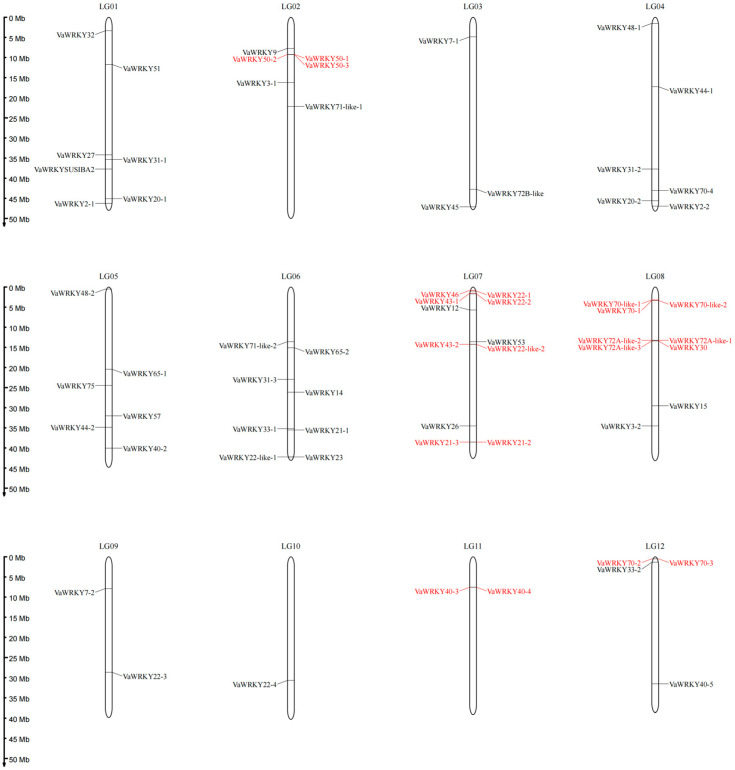
The distribution of *VaWRKY* family members on the chromosomes of *V. bracteatum*. The gene names on both sides of each chromosome correspond to the approximate locations of each *VaWRKY* gene, with the left scale measured in megabases. Genes showing gene clustering are indicated in red.

Through whole-genome chromosome mapping analysis, this study identified 65 *VaWRKY* genes distributed across 12 chromosomes in the *VaWRKY* genome, with *VaWRKY40-1* located on chromosome Contig00366 (Figure 5). The genes are mainly concentrated on chromosomes 1, 6, 7, and 8 (with at least seven genes), while chromosome 10 has the fewest genes, containing only one gene. Notably, visual inspection of the *VaWRKY* gene locations reveals regions with higher *VaWRKY* gene density, known as “clusters” of *WRKY* genes. Gene clustering is observed on chromosomes 2, 7, 8, 11, and 12. The closer two genes are to each other on a chromosome, the lower the probability of recombination between them, indicating a stronger linkage and a higher likelihood of being inherited together. These clustered genes may also expand through tandem duplication mechanisms, suggesting that they have undergone positive selection during evolution to adapt to specific stress environments.

**Figure 6:**
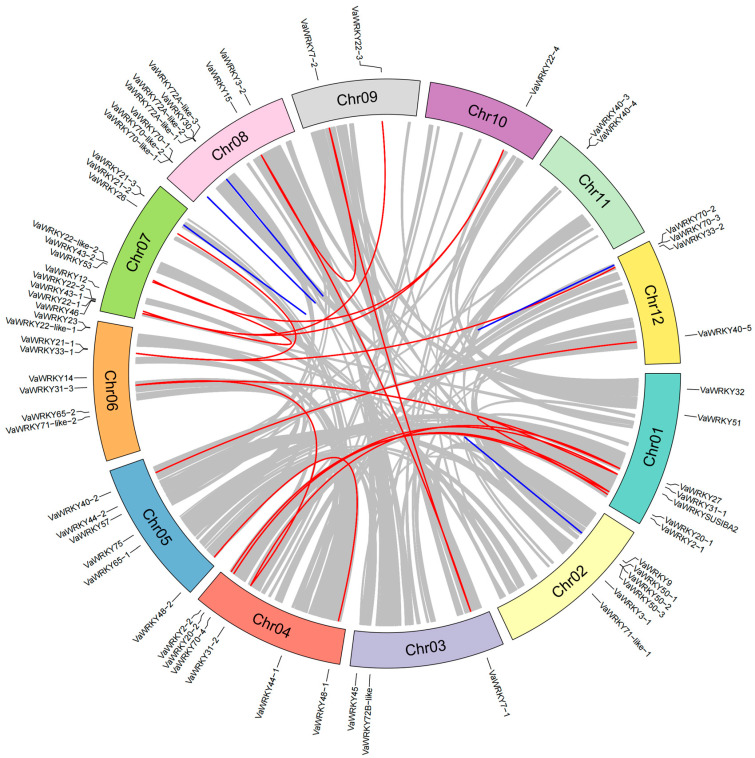
Co-linearity analysis of the *VaWRKY* gene family. The relative positions of the genes are shown on the 12 chromosomes. The gray line represents the collinearity of the entire genome. The blue line represents tandem repeats, and the red line represents segmental duplications.

Two homologous repeats within the same chromosome that are less than 200 kb apart are usually referred to as tandem repeats. To investigate the gene duplication events, we performed collinearity analysis of the *VaWRKY* gene family by using BLASTp and MCScanX (Figure 6). The results are shown in the figure. We observed five pairs of tandem duplications involving 10 *VaWRKY* genes, with all tandem duplication pairs belonging to the same subgroup. Meanwhile, we identified 18 syntenic gene pairs involving 24 *VaWRKY* genes, where some genes participated in multiple pairing relationships, suggesting that certain genes may have undergone more than one duplication event.

Synonymous mutation (Ks) and non-synonymous mutation (Ka) are usually used to verify the selection pressure. All duplicated gene pairs in *VaWRKY* had Ka/Ks ratios below 1. This means that these gene pairs underwent purifying selection. Notably, the *VaWRKY50-2*/*VaWRKY50-3* gene pair was excluded from this analysis potentially due to its substantial length divergence, despite displaying high sequence similarity, which precluded reliable calculation of Ka/Ks ratios. These findings suggest that tandem duplication and segmental duplication events have played distinct roles in the expansion of the *VaWRKY* family, with purifying selection acting as a critical force in maintaining functional conservation among duplicated genes.

### 2.6. Promoter and Homology Analysis of the WRKY Gene Families in V. bracteatum

**Figure 7:**
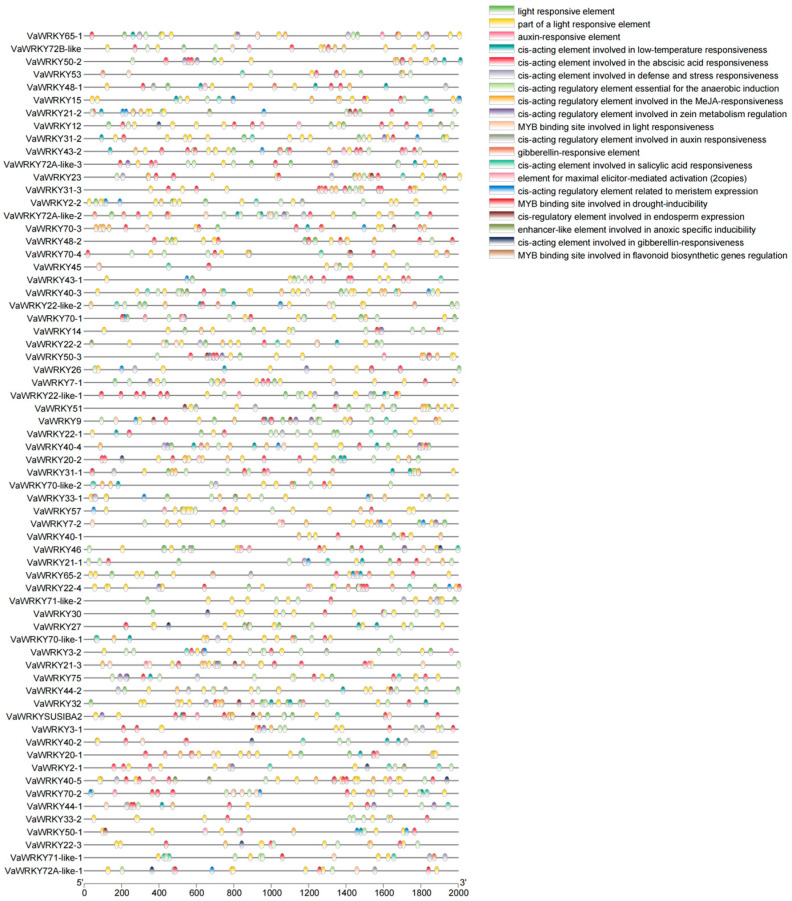
Analysis of cis-regulatory elements in the *VaWRKY* gene family. Regulatory elements found in the promoter region are represented by blocks of different colors.

According to the prediction results from PlantCARE, 1684 cis-acting elements of interest were identified (Figure 7). Among these, 744 elements are light-responsive or involved in light response, almost all of which are present in the promoters of *VaWRKY* genes, with *VaWRKY40-5* being the most abundant at 21 elements and *VaWRKY40-1* the least at only four elements. This suggests that light signals may have broad regulatory effects on members of the *VaWRKY* gene family. There are also 512 hormone-responsive or hormone-interacting elements, with the highest number being those involved in ABA and MeJA responses, totaling 431 elements. Other hormone-related elements include those involved in auxin, gibberellin, and salicylate responses. The promoter regions of *VaWRKY* genes also contain numerous elements related to stress responses, including those associated with anaerobic induction, drought induction, cold response, and defense and stress responses. Additionally, some elements related to plant growth and development have been found upstream of *VaWRKY* genes. These elements are diverse, suggesting that these genes may play roles in multiple aspects of plant growth and development.

### 2.7. Expression Pattern Analysis of VaWRKY

**Figure 8:**
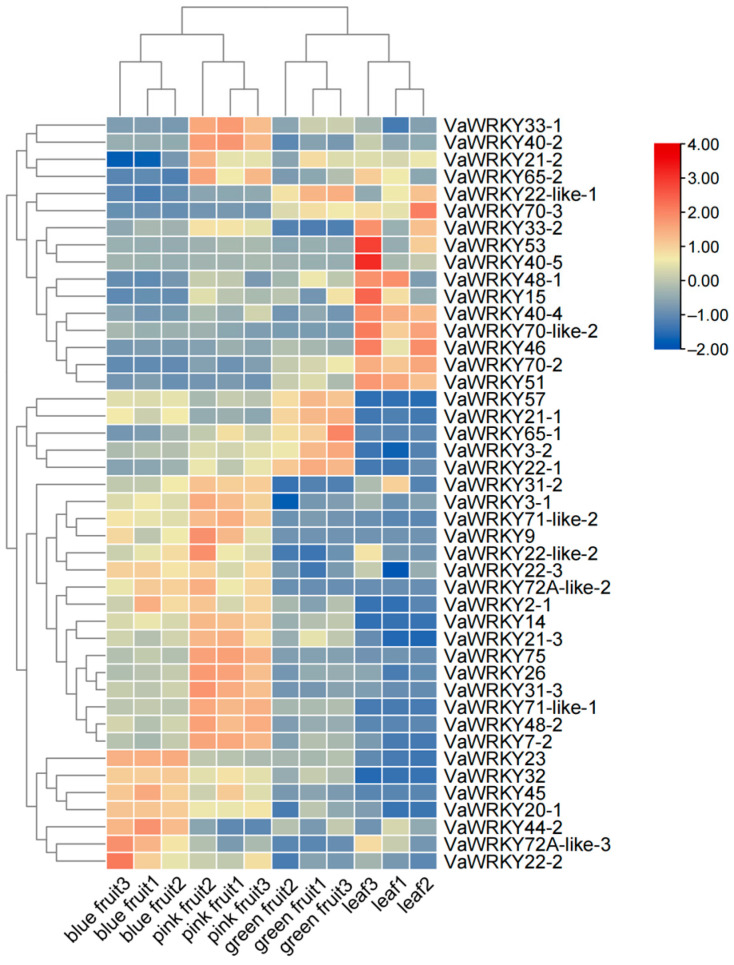
Expression pattern of *WRKY* gene in various tissues of *V. bracteatum*. Each column in the figure represents the expression of different genes at the same stage, and each row represents the expression of the same gene at different stages. From blue to red, you can see that the value gradually increases.

To explore the potential functions of the *VaWRKY* gene in the growth and development of *V. bracteatum* and to further investigate the contemporary roles and importance of *WRKY* transcription factors, we obtained gene expression data at different fruit stages through transcriptome sequencing (RNA-Seq). The reliability of the transcriptome data was further validated by quantitative real-time PCR (qRT-PCR) experiments (Figure S2). We used the HeatMap function in TBtools v2.083 software to generate cluster heatmaps to analyze the expression patterns of *WRKY* genes (Figure 8). During the fruiting period of *V. bracteatum*, we collected samples from three developmental stages: green-fruit stage (immature), pink-fruit stage (intermediate), and blue-fruit stage (mature). Samples were repeatedly collected from three individual plants at each stage and stored in liquid nitrogen for subsequent transcriptomic analysis. In addition to the green, pink, and blue fruits of *V. bracteatum*, the materials also included leaves of *V. bracteatum*. Based on the expression analysis, we found that among the *VaWRKY* gene family, 12 *WRKY* genes exhibited significant expression differences at different stages of fruit maturation (green, pink, blue fruits), with most *VaWRKY* genes showing higher expression levels in pink fruits. Furthermore, the expression analysis results revealed specific patterns of upregulation or downregulation of *WRKY* genes in *V. bracteatum* during fruit maturation. Most *WRKY* genes showed a significant increase in expression levels during the later stages of fruit maturation (blue-fruit stage), such as *VaWRKY23* and *VaWRKY32*. Some genes (such as *VaWRKY22-like-1* and *VaWRKY57*) are highly expressed in the green fruit stage, and their expression gradually decreases with the maturation of fruit. Some genes are only upregulated in specific stages (such as blue fruit), indicating that they may play a key regulatory role in fruit maturation.

In the *WRKY* gene of the *V. bracteatum*, the *VaWRKY* gene in cluster 1 is expressed higher in leaves than during fruiting, suggesting that it may play a role in leaf development. In contrast, cluster 2 shows almost no expression in leaves but is significantly and markedly expressed during fruiting, indicating its potential role in fruit development. Additionally, 12 genes exhibit significant differential expression during fruit maturation, suggesting their involvement in fruit development (Table S2). Interestingly, *VaWRKY22-like-1*, *VaWRKY57*, *VaWRKY21-1*, *VaWRKY65-1*, *VaWRKY3-2*, and *VaWRKY22-1* are highly expressed during green-fruit stages, indicating that these genes may be related to fruit enlargement; while *VaWRKY23*, *VaWRKY32*, *VaWRKY45*, *VaWRKY20-1*, *VaWRKY44-2*, *VaWRKY72A-like-3*, and *VaWRKY22-2* are highly expressed during blue-fruit stages, suggesting that they may be associated with fruit ripening, making them valuable for research. Particularly noteworthy are *VaWRKY22-4* (homologous to *AtWRKY27*, possibly involved in disease resistance regulation) and *VaWRKY50-1* (contains a zinc finger domain).

### 2.8. Analysis of Protein Interaction Networks

**Figure 9:**
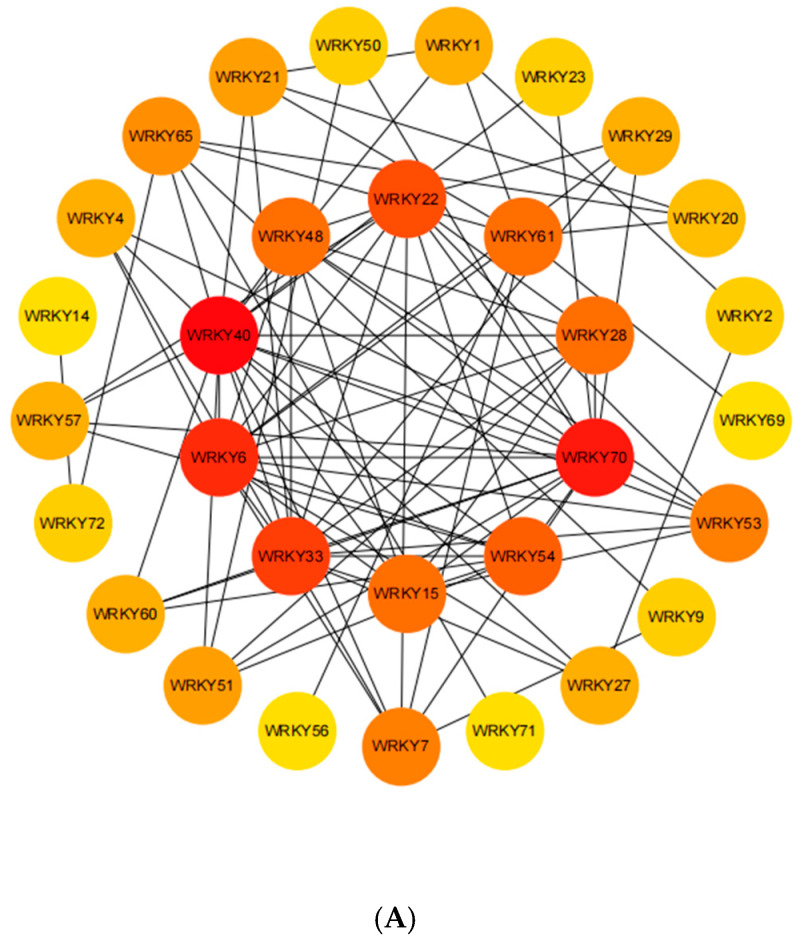

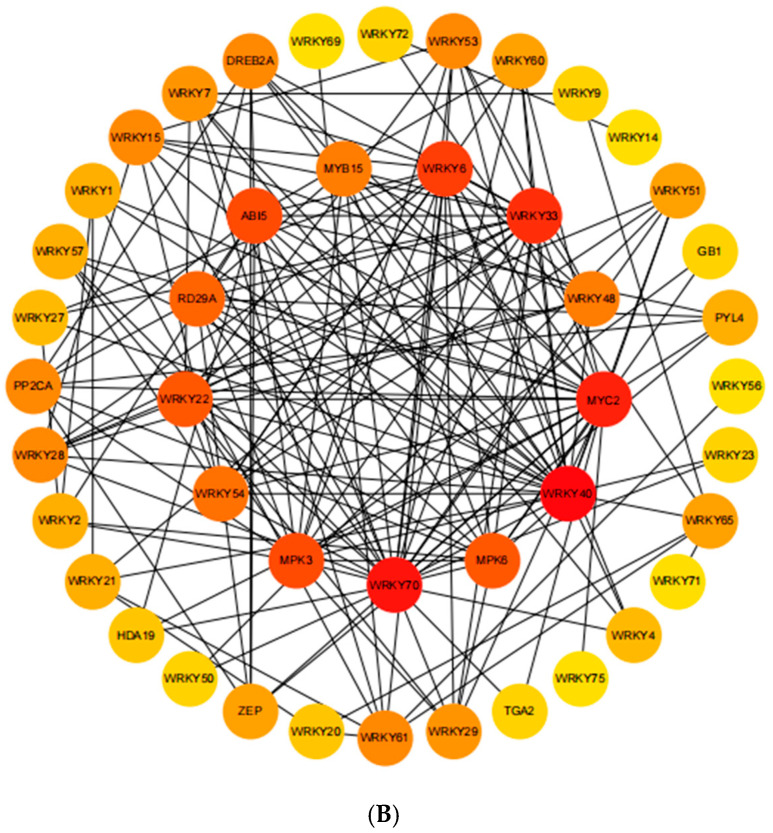
Predicted *VaWRKY* gene family protein interaction analysis. (**A**) The internal interactions of *VaWRKY* transcription factors. (**B**) Interactions between *VaWRKY* proteins and other functional proteins. The orange-yellow ball (node) represents the *VaWRKY* gene, and the thickness of the connecting line represents the correlation between the two proteins.

To predict the biological functions and potential regulatory roles of *VaWRKY* protein, we used the STRING database based on data from *AtWRKY* protein interactions and constructed a *VaWRKY* protein interaction network using Cytoscape v3.10.3 software (Figure 9A). The protein-protein interaction analysis network indicates that the *VaWRKY* protein, which is highly similar to the *AtWRKY40* (*VaWRKY40*), *AtWRKY70* (*VaWRKY70-1*, *VaWRKY70-2*, *VaWRKY70-3*, *VaWRKY70-like*), *AtWRKY6* (*VaWRKY31*), *AtWRKY33* (*VaWRKY33*, *VaWRKY26*), and *AtWRKY22* (*VaWRKY22-1*, *VaWRKY22-2*, *VaWRKY22-3*, *VaWRKY27*) sequences, is a key protein in the protein-protein interaction network. Other proteins have varying degrees of interaction with these proteins. These transcription factors specifically interact with the W-box (5′-(T)TGAC[CT]-3′), a common cis-acting element in promoter regions. Through similarity comparisons, these transcription factors may play roles in regulating various plant hormone signaling pathways, participating in plant growth and development processes, as well as responses to biotic and abiotic stresses.

To gain a deeper understanding of the biological functions of the *VaWRKY* gene, we analyzed the protein-protein interactions between *VaWRKY* and other functional genes (Figure 9B). As we anticipated, the functions of the *VaWRKY* gene are predominantly enriched in stress responses, seed germination regulation, and plant growth and development regulation. For example, *VaWRKY* interacts with DRE binding proteins, ABI (ABA-insensitive) proteins, mitogen-activated protein kinase MAPK, MYC proteins, and MYB proteins, collaborating to regulate plant growth and development or various stress responses.

### 2.9. Three-Dimensional Structure Analysis of WRKY Protein in V. bracteatum

To understand the structural characteristics of *VaWRKY* proteins, we used the amino acid sequences of 66 *VaWRKY* proteins to predict their 3D structure models through the SWISS Model website (Figure S1). The helical structure is the α helix, the parallel lamellar structure is β fold, and the point is zinc ion. Ramachandran plot is used to verify the models of various obtained proteins. In the 3D structures of these 66 *VaWRKY* proteins, most contain three specific structures: α helices, β sheets, and random coils. Only two *VaWRKY* proteins (*VaWRKY40-1* and *44-2*) have distinct zinc ion structures, known as zinc finger domains, which may indicate zinc-dependent proteins. Differences in protein structure have extensive impacts on protein sequence, stability, and activity. These structural differences can provide structural diversity and functional versatility to *VaWRKY* proteins.

## 3. Discussion

*V. bracteatum* is a wild blueberry species belonging to the *Vaccinium* genus. It is an evergreen shrub or small tree widely distributed in hilly areas of China [19,20]. *V. bracteatum* is a kind of plant integrating multiple functions, such as medicine, food, and ornamental. It is widely cultivated in China. As a native plant, it can resist the impact of many adverse factors and has high research value.

The *WRKY* gene family is an important class of transcription factors in plants, Family members appear to be involved in the regulation of various physio-logical programs that are unique to plants, including pathogen defense, senescence, trichome development [21], and a variety of biological functions. With the increasing attention to various cash crops and the development of technologies such as genome identification, more and more *WRKY* gene families have been identified in plants, such as asparagus (*Asparagus officinalis*) [22], pea (*Pisum sativum*) [23], eggplant (*Solanum melongena*) [24], and peanut (*Arachis hypogaea*) [25], but there are few studies on the full genome identification and analysis of *WRKY* in a high economic native wild blueberry species in China that has not been fully utilized.

To fill the gap in research on the *WRKY* gene family in *V. bracteatum*, this study utilized various online network tools and genomic sequencing data to comprehensively identify and analyze *WRKY* transcription factors in *V. bracteatum*. This includes the identification of the *VaWRKY* gene family, phylogenetic tree analysis, chromosome mapping and collinearity analysis, gene structure and conserved domain analysis, cis-regulatory element analysis, expression analysis of *VaWRKY* during fruit maturation, protein-protein interaction analysis, and three-dimensional structure analysis.

### 3.1. Analysis of the Evolutionary Trajectory and Structural Characteristics of the VaWRKY Gene Families

This study identified and screened out 66 *VaWRKY* genes from the whole genome of *V. bracteatum*, with a higher number of genes than pineapple (*Ananas comosus*) (54 members) [26], *Trifolium pratense* (59 members) [18], but much lower than weeping forsythia (*Forsythia suspensa*) (79 members) [27], cabbage (*Brassica oleracea*) (105 members) [28], and cotton (*Gossypium hirsutum*) (116 members) [29]. We already know that the abundance of transcription factor gene families largely depends on sequence duplication events during genome evolution [30]. The relatively low number of *VaWRKY* genes in *V. bracteatum* suggests that gene duplication and deletion may have occurred during genome evolution. Additionally, this study predicted the physicochemical properties of candidate *VaWRKY* family members, including protein molecular weight, isoelectric point, and subcellular localization, using online websites such as ExPASy, and Plant-mPLoc, to better predict the function and structure of *VaWRKY* proteins.

To reveal the evolutionary relationships and species divergence history among *VaWRKY* genes, this study conducted a phylogenetic analysis on 66 *VaWRKY* genes and 72 *AtWRKY* genes. Through multiple sequence alignment, the 66 *VaWRKY* proteins were divided into three groups: I, II, and III. In terms of quantity, Group III contains the most genes, with 28 members, accounting for about 42% of the total. Additionally, by examining the distribution of *VaWRKY* in each sub-group, we infer that the expansion event of the *WRKY* family occurred before the divergence of snapdragon and *A. thaliana*. The *WRKY* transcription factor family includes a highly conserved WRKYGQK heptapeptide sequence [31], with Group I comprising 14 members, most of which contain two *WRKY* domains. However, some *VaWRKY* proteins contain only one *WRKY* domain, such as *VaWRKY50-1*, *44-1*, and *44-2*, a phenomenon also observed in cassava (*Manihot esculenta*) [32] and *A. thaliana* [21]. Meanwhile, some *VaWRKY* proteins have undergone mutations in their WRKYGQK heptapeptide domains, with Q mutations to K, E, and R forming WRKYGKK, WRKYGEK, and WRKYGRK structures. This change may affect the interaction between *WRKY* genes and downstream target genes, indicating that *VaWRKY* deserves further investigation into its functional specificity [33,34].

Analyzing the distribution of introns and exons in the genome is crucial for understanding the structure and function of genes, as the diversity of intron-exon structures is a significant component of gene family evolution and can effectively illustrate the diversity of *VaWRKY* functions [35]. In this study, we found that 66 *VaWRKY* genes contain 1 to 11 exons and 0 to 10 introns, with *VaWRKY* genes within the same branch exhibiting similar exon-intron structures. *VaWRKY27* is a unique presence, being the only member without introns and having the shortest gene length. *VaWRKY27* does not express effectively in the *V. bracteatum* tissue we studied. *VaWRKY33-1* has the highest number of exons and introns and the longest gene length. Analysis of cis-regulatory elements reveals that it contains multiple cis-regulatory elements, indicating its important role in expression regulation. The high ratio of introns to exons suggests that *WRKY* genes exhibit high conservation during evolution, suggesting their significant function in adaptation and survival. To study the structural diversity among *WRKY* gene families in the *V. bracteatum*, we conducted a conservative motif prediction for *VaWRKY* proteins. Among them, motif1 and motif3 include the *WRKY* heptapeptide structure, while motif2 contains a zinc finger domain. Through multiple sequence alignment, we found that all 64 *VaWRKY* gene proteins, except for genes *VaWRKY50-1* and *VaWRKY50-2*, contain motif1. This indicates that motif1 plays a crucial structural role in *WRKY* transcription factors and is one of the core conserved domains. This discovery further reinforces the importance of *WRKY* transcription factors as regulators of gene expression in plant biology. However, genes *VaWRKY50-1* and *VaWRKY50-2* do not contain motif1, suggesting that these genes may be functionally redundant or that they do not perform conventional functions in *V. bracteatum*. Additionally, these unique gene compositions may be the primary reason for the classification and evolution of *WRKY* genes in *V. bracteatum*. To further investigate this hypothesis, we can validate the specific functions and biological significance of these genes through gene knockout or overexpression experiments in future research. We found through our analysis that the conserved motifs motif1 and motif2 always appear in pairs, which may indicate a functional association between these two motifs or suggest that they need to coexist to exert certain functional roles. We speculate that motif1 and motif2 might have a synergistic effect on the function of the *WRKY* gene protein in the *V. bracteatum*. To verify this hypothesis, we will design double mutant experiments to observe the impact of the absence of these two motifs on the protein’s function.

In the *V. bracteatum*, the domains present in the *WRKY* gene family are diverse. In addition to the common *WRKY* domain, many *WRKY* genes also have other domains, and these domains exhibit varied functions. This suggests that the *WRKY* gene may be involved in multiple physiological processes during the growth and development of the *V. bracteatum*. Among them, the N-terminal domain of the *WRKY* domain in *VaWRKY50-1* of Group I is incomplete, while the C-terminal domain of the *WRKY* domain in *VaWRKY44-2* is incomplete. According to early studies, only the C-terminal domain of Group I has sequence-specific DNA binding activity with W-box, whereas the N-terminal *WRKY* domain has weaker binding activity [36]. The N-terminal *WRKY* domain can provide an interface for protein-protein interactions that matches the function of certain zinc finger-like domains [37]. Therefore, it is inferred that the absence of either end of the *WRKY* domain could significantly impair the transcription factor’s function, but this requires experimental verification.

### 3.2. Chromosomal Distribution Pattern and Gene Duplication Mechanism of the VaWRKY Gene Families

Gene duplication events drive species adaptive development through a series of genome rearrangements and expansions, serving as a crucial mechanism for gene evolution [38]. Segmental duplications, tandem duplications, and transposons are the three primary modes of gene family expansion [39]. To elucidate the evolutionary mechanisms of the *WRKY* gene family in *V. bracteatum*, we conducted chromosomal mapping and synteny analysis of *VaWRKY* genes. *V. bracteatum* is a diploid plant with 12 chromosomes. We identified 65 *VaWRKY* genes distributed across these 12 chromosomes, while *VaWRKY40-1* is located on chromosome Contig00366. The number of genes on each chromosome varies, with chromosome 7 having the most genes and chromosome 10 having the fewest, containing only one gene (*VaWRKY22-4*). This uneven gene distribution and evidence of gene clustering and duplication provide valuable insights into the genomic organization and evolutionary dynamics of the TFs gene families in *V. bracteatum* [40]. In addition, gene clustering occurs on some chromosomes, with chromosomes 7 and 8 showing the most pronounced pattern. The closer two genes are to each other on a chromosome, the lower the probability of recombination between them and the tighter their connection. This may suggest that these closely related genes might need to interact to perform a life activity of the *V. bracteatum*. Therefore, they are generally inherited together.

### 3.3. Construction of Cis—Elements in the VaWRKY Gene Promoter and Expression Regulation Network

Previously, many studies have shown that *WRKY* transcription factors play a crucial role in plant stress resistance and are also involved in growth regulation of the plant [41]. In addition, researchers have hypothesized that the *WRKY* gene family participates directly or indirectly in the growth and development of flowers, fruits, and seeds through responses to auxins, gibberellins, salicylic acid, and methyl jasmonate. Analysis of the expression levels of the *WRKY* gene family at different stages in the *V. bracteatum* indicates that most members of the *WRKY* gene family increase in expression during fruit maturation in red fruits, while they gradually decrease in other periods. The expression levels of corresponding family members vary across different stages, suggesting that the *WRKY* gene family can regulate fruit maturation and color transformation. The expression profile of *WRKY* genes provides insight into their potential functions. Most *WRKY* genes in the *V. bracteatum* are highly expressed during the maturation stage, with lower expression levels during leaf and green fruit development stages, indicating that these genes may play important roles in fruit maturation, possibly performing additional necessary activities for fruit formation. Previous studies have found that 33% of *ClWRKY* genes are expressed in the fruit of watermelon, some of which are positively or negatively involved in plant hormone regulatory pathways and play significant roles in plant growth, development, and resistance to environmental stresses [42]. In bananas, some *MaWRKYs* are highly expressed in mature fruits, while others are lowly expressed during fruit maturation. *MaWRKY49* directly regulates the expression of pectin lyase, serving as a potential regulatory factor for fruit maturation [14], indicating that *MaWRKYs* play different roles in fruit development and maturation [43]. In our study, most *WRKY* genes are also highly expressed during fruit maturation, suggesting that some *WRKY* genes may be involved in plant hormone regulation pathways, playing a crucial role in plant growth, development, and stress resistance. Additionally, the family of these genes exhibits specific expression at different stages, indicating possible specialization within the gene family.

Research on the promoter elements of the *VaWRKY* gene family can help us understand the molecular regulatory mechanisms of *WRKY* transcription factors in plants and also provide theoretical basis for improving crop stress resistance and efficient cultivation. We extracted and comprehensively analyzed the 2kb upstream region of the *WRKY* gene in *V. bracteatum*, finding that the promoter sequence of the *VaWRKY* gene is distributed with various cis-acting elements, such as light-responsive elements, hormone-responsive elements, and elements related to stress responses. This indicates that the regulatory mechanisms of *WRKY* genes in *V. bracteatum* are diverse. Among these, the widespread presence of light-responsive elements (744) and plant hormone-responsive elements (512) suggests that *WRKY* genes may be involved in plant growth, development, and hormone signaling pathways. This promoter analysis reveals that *WRKY* genes play a complex regulatory network and significant role in plant life activities.

### 3.4. Analysis of the VaWRKY Protein Interaction Network and Functional Prediction

At the protein level, *WRKY* transcription factors can interact with various proteins, including MAP kinases, MAPK kinases, VQ cofactor, and multiple transcription factors, regulating plant growth and development or various stress responses. To understand the biological functions and regulatory networks of *WRKY* proteins in *V. bracteatum*, we constructed a *VaWRKY* protein interaction network. As previously discovered, the functions of *VaWRKY* genes are mainly reflected in stress response, seed germination regulation, and plant growth and development regulation. Among these, *WRKY33* interacts most closely with other functional proteins, and *WRKY33* transcription factors are highly conserved during the course of plant evolution not only in structure and function but also in the regulatory network [44]. Therefore, the *WRKY33* pathway of the *V. bracteatum* is highly conserved, protecting it from many important biological and abiotic stresses.

In addition, we used the Swiss Model online tool to predict the three-dimensional structural models of the *WRKY* protein family, aiming to explore the structural basis for the functional differentiation of *WRKY* proteins in the *V. bracteatum* and to elucidate the DNA binding specificity and molecular mechanisms. The three-dimensional structural model of *VaWRKY* protein reflects the diversity of *WRKY* protein structures, which also determines the specificity of protein functions. This diversity also demonstrates the high adaptability and regulatory capabilities of living systems, providing crucial theoretical evidence for protein engineering and the improvement of crop stress resistance.

## 4. Materials and Methods

### 4.1. Plant Resources

Specimens of the *V. bracteatum* were collected in the subtropical hilly region of southern Anhui (30°51′ N, 118°23′ E) to obtain basic genomic data for this species. The genome data and protein sequences of *A. thaliana* were downloaded from the TAIR website (https://www.arabidopsis.org/) (accessed on 3 December 2024). During the vegetative growth of the *V. bracteatum*, leaves and fruits at different developmental stages were collected as materials. Green fruits (S1), pink fruits (S2), and mature blue fruits (S3) were collected during the fruiting period. Samples were taken from three individual plants at each developmental stage and prepared into three test tubes. The samples were then rapidly frozen in liquid nitrogen and stored at −80 °C in an ultra-low temperature freezer for future research. RNA was extracted from the samples, and the obtained RNA samples were used for transcriptome gene prediction. All the samples were processed using the mirVana miRNA Isolation Kit (Ambion) following the manufacturer’s protocol. Sequencing libraries were generated using NEBNext Ultra^TM^ RNA Library Prep Kit for Illumina (New England Biolabs, Ipswich, MA, USA) following the manufacturer’s instructions, and index codes were added to attribute sequences to each sample. The clustering of the index-coded samples was performed on a cBot Cluster Generation System, using TruSeq PE Cluster Kit v4-cBot-HS (Illumina) according to the manufacturer’s instructions. After cluster generation, the library preparations were sequenced on an Illumina platform, and paired-end reads were generated at BIOMARKER (Beijing, China) [45]. Sequencing data used in this study are available in the NCBI Sequence Read Archive (SRA) database under the following accession numbers: BioProject PRJNA794927.

### 4.2. Genome-Wide Identification and Classification of WRKY Gene Families

The Hidden Markov Model (HMM) files for the *VaWRKY* domain (PF03106) used in this study were obtained from the Pfam database (http://pfam.xfam.org/) (accessed on 5 December 2024). Using this model, the HMMER3.3.2 software (http://hmmer.org/) (accessed on 5 December 2024) was employed to perform a whole-genome scan of the *V. bracteatum* genome, screening for candidate proteins containing the *WRKY* domain. Through the aforementioned HMMER screening, 66 proteins with complete *WRKY* domains were identified in the *V. bracteatum* genome.

Using the seed tool BLASTp from the National Center for Biotechnology Information (NCBI) database (https://www.ncbi.nlm.nih.gov/) (accessed on 8 December 2024), *VaWRKY* gene features were compared online and assigned specific names (Table S3). The website ExPASy (https://web.expasy.org/protparam/) (accessed on 8 December 2024) was used to predict the protein molecular weight (MW), isoelectric point (pI), amino acid length, total number of negatively charged residues, total number of positively charged residues, instability index, aliphatic amino acid index, and average hydrophobicity (grand average of hydropathicity, GRAVY) of candidate *VaWRKY* family members. Additionally, the Plant-mPLoc website (http://www.csbio.sjtu.edu.cn/bioinf/plant-multi/) (accessed on 8 December 2024) was used to predict the subcellular localization of *VaWRKY* genes.

### 4.3. The Establishment of the Phylogenetic Tree

The *WRKY* genome data of *V. bracteatum* used in the article was obtained through laboratory analysis, while the *A. thaliana* genome data and protein sequences were acquired from the TAIR website (https://www.arabidopsis.org/) (accessed on 12 December 2024). The online software ClustalX2.1 was used to construct phylogenetic trees for *V. bracteatum* and *A. thaliana* using the minimum-branching method (NJ), with bootstrap set to 1000 and other parameters left at default values. The complete phylogenetic tree was drawn using the R: ggtree package. All identified *WRKY* genes of *V. bracteatum* were classified into different groups based on the *AtWRKY* classification scheme and the alignment of *VaWRKY* and *AtWRKY* proteins.

### 4.4. Analysis of Introns, Exons, and Regulatory Sequences

Using the online program Gene Structure Display Server GSDS 2.0 (http://gsds.gao-lab.org/) (accessed on 15 December 2024), the *VaWRKY* GFF3 annotation file format for genomic sequences and coding sequences (CDS) was uploaded and matched to obtain the gene structure of *VaWRKY* genes, thereby illustrating the exon-intron structure diagram and coding sequences (CDS) configuration of *VaWRKY* genes. The online database MEME (https://meme-suite.org/meme/tools/meme) (accessed on 15 December 2024) was used to analyze the conserved base sequences of all *WRKY* proteins in *V. bracteatum*, with the maximum conservation motif search value set to 10 and other parameters kept at default values, saving both MAST XML output files and MEME HTML output files. It was visualized using TBtools v2.083.

### 4.5. Analysis of Related Structural Domains

To elucidate the conserved characteristics and functional differentiation mechanisms of *VaWRKY* gene family proteins, we used NCBI Batch CD-Search (https://www.ncbi.nlm.nih.gov/Structure/bwrpsb/bwrpsb.cgi) (accessed on 17 December 2024) to perform domain scanning of *VaWRKY* family proteins. We selected default parameters and focused on the signature domains of the *VaWRKY* gene family, downloading the results in txt format. Then, using the Gene Structure View function in TBtools v2.083, we input the predicted domain txt files from NCBI and the amino acid sequences of the *VaWRKY* family to generate domain distribution pattern diagrams.

### 4.6. Visualization and Collinearity Analysis of Chromosomal Positioning

Based on the latest version of the *V. bracteatum WRKY* genome annotation file (GFF3 format) and the chromosomal location information of all *WRKY* genes, including gene ID, chromosome number, and start and end coordinates, we used the MG2C online website (http://mg2c.iask.in/mg2c_v2.1/) (accessed on 21 December 2024) to map the chromosomal distribution of *WRKY* genes in *V. bracteatum*. Different colors were used to mark the positions of tandem repeat gene clusters, providing an intuitive display of the distribution characteristics of *VaWRKY* gene family members across various chromosomes. To investigate the mechanisms of *WRKY* gene family expansion, we used the BLASTp tool to compare whole-genome protein sequences, inputting the comparison results into MCScanX v1.1 for co-linearity analysis of the *VaWRKY* gene family. We also validated selection pressure using synonymous mutations (Ks) and non-synonymous mutations (Ka) for the detected co-linear *VaWRKY* genes.

### 4.7. Analysis of Promoter Sequence

To investigate the potential regulatory elements and their functions in the promoter regions of *VaWRKY* gene family members, this study used TBtools v2.083 to extract the upstream 2000 base pairs of the *VaWRKY* gene family from the genome sequence of *V. bracteatum* as promoter sequences. The regulatory elements in the promoter region were further analyzed using PlantCARE (https://bioinformatics.psb.ugent.be/webtools/plantcare/html/) (accessed on 30 December 2024). To minimize false positives, only elements that have been repeatedly reported and whose functions are clearly defined in the PlantCARE annotation database (repeated times ≥ 2) were retained. Ultimately, TBtools v2.083 was used to classify these elements based on their biological functions into hormone-responsive, light-responsive, and tissue-specific expression-related elements. The distribution characteristics of these elements in different gene promoter regions were visualized to reveal their potential regulatory mechanisms.

### 4.8. Expression Patterns Within Different Fruit Stages

During the fruiting period of the *V. bracteatum*, we collected samples from three stages: green fruits, pink fruits, and blue fruits. Additionally, we gathered leaf samples from the *V. bracteatum*. Samples were repeatedly taken from three individual plants at each stage and prepared into three replicates. Full RNA was precisely isolated from a total of 12 samples. To explore the potential functions and significant roles of *WRKY* genes in the growth and development of the *V. bracteatum*, we used data from *VaWRKY* gene family expression after transcriptome analysis. Using the HeatMap function in TBtools v2.083, we generated cluster heatmaps to display the expression patterns of *VaWRKY* genes and analyze differential expression of *WRKY* genes in different tissues and at different times within the same tissue.

### 4.9. Analysis of Protein–Protein Interaction Expression

Based on the amino acid sequence of *VaWRKY* protein, using the STRING database (http://string-db.org/) (accessed on 13 January 2025), species was set to *A. thaliana* for interaction prediction, with a confidence threshold of 0.4 (medium confidence). Hidden inactive proteins were excluded, and the string_interactions.tsv file was downloaded. Then, using the Cytoscape software v3.10.3, the interaction relationships output by STRING (in tsv format) were visualized. Through the CytoHubba plugin, the Drgree algorithm was selected, and the top 31 proteins ranked according to MCC were chosen as key proteins for further study.

### 4.10. Protein 3D Structure Analysis

To elucidate the spatial conformational characteristics of *VaWRKY* gene family proteins, we used TBtools v2.083 to annotate the *VaWRKY* gene sequences into amino acid sequences and save them in FASTA format. We then utilized the SWISS Model website (https://swissmodel.expasy.org/) (accessed on 15 January 2025) as a template to predict the three-dimensional structure of *VaWRKY* proteins. The model quality was evaluated using Global Model Quality Estimation (GMQE) and QMEAN values. Finally, the predicted model images were beautified and stitched together using Adobe Illustrator CS6 software.

## 5. Conclusions

In this study, 66 *VaWRKY* genes were identified from the genome of *V. bracteatum*. We utilized various online tools and obtained genomic and expression data from *V. bracteatum* RNA samples for whole-genome identification, phylogenetic analysis, gene structure and conserved motifs, chromosomal location, synteny, promoter regions, expression during fruit maturation, protein interactions, and three-dimensional protein structure analysis. This provides comprehensive information on the *WRKY* gene family in *V. bracteatum*, distinguishing the composition, evolutionary relationships, and functions of *WRKY* family genes in fruit maturation. It serves as a guide for molecular breeding in *V. bracteatum* and offers theoretical support for future research on stress tolerance mechanisms and genomics in *V. bracteatum*.

## Data Availability

The original contributions presented in the study are included in the article/Supplementary Materials; further inquiries can be directed to the corresponding authors.

## Supplementary Materials

The following supporting information can be downloaded at https://www.mdpi.com/article/10.3390/ijms26167835/s1.

## Abbreviations

The following abbreviations are used in this manuscript:

NJ	neighbor-joining
ABA	abscisic acid
MeJA	methyl jasmonate
DRE	defective in RNA polymerase II transcription factor D
MAPK	mitogen-activated protein kinase
MAP	microtubule-associated proteins
