# Supplementary material for: Genome-Wide Identification and Expression Analysis of the WRKY Gene Families in Vaccinium bracteatum
Source: Int J Mol Sci. 2025 Aug 13;26(16):7835. doi: 10.3390/ijms26167835 (PMC12386343; doi:10.3390/ijms26167835)
Supplement: Supplementary file 1 [file ijms-26-07835-s001.zip › Figure S1 3D structure model of 66 VaWRKY proteins.pdf]

Figure S1 3D structure model of 66 *VaWRKY* proteins

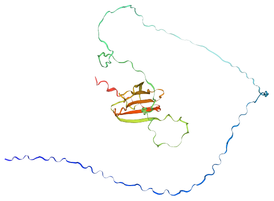

VaWRKY12

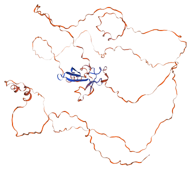

VaWRKY14

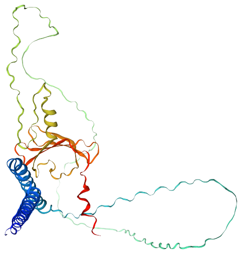

VaWRKY15

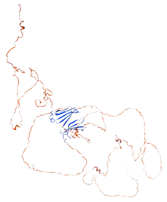

VaWRKY2-1

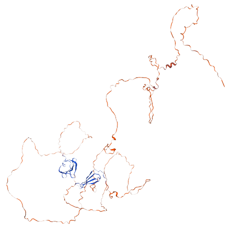

VaWRKY2-2

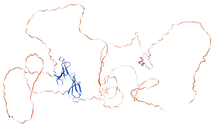

VaWRKY20-1

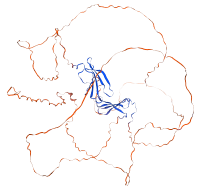

VaWRKY20-2

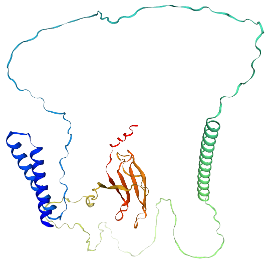

VaWRKY21-1

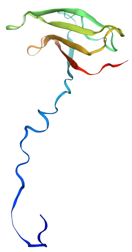

VaWRKY21-2

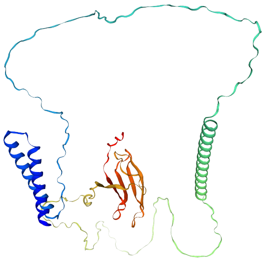

VaWRKY21-3

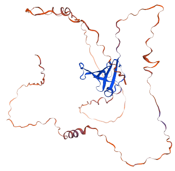

VaWRKY22-1

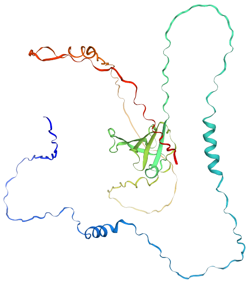

VaWRKY22-2

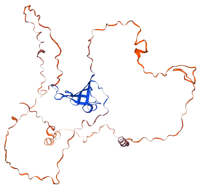

VaWRKY22-3

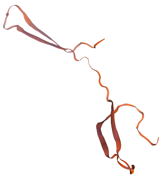

VaWRKY22-4

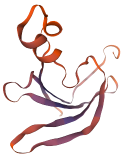

VaWRKY22-like-1

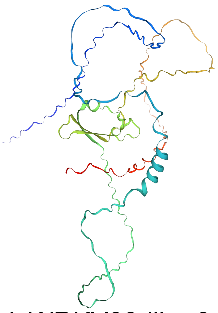

VaWRKY22-like-2

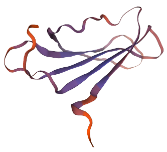

VaWRKY23

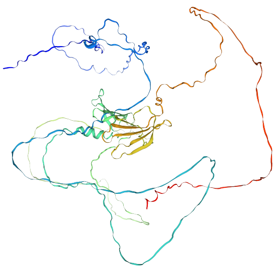

VaWRKY26

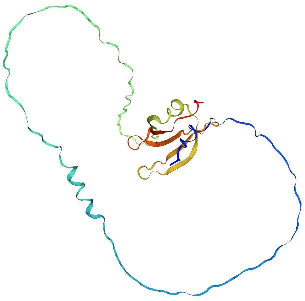

VaWRKY27

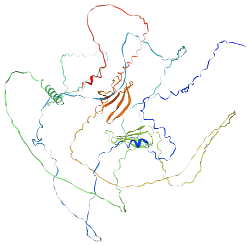

VaWRKY3-1

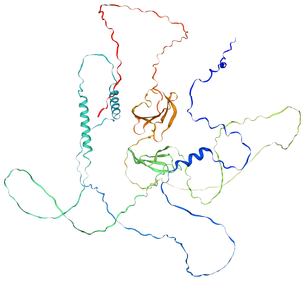

VaWRKY3-2

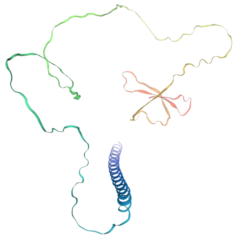

VaWRKY30

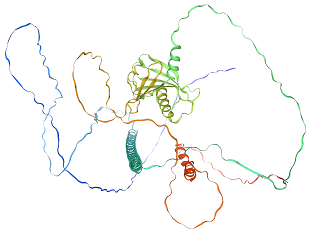

VaWRKY31-1

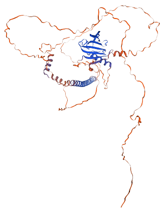

VaWRKY31-2

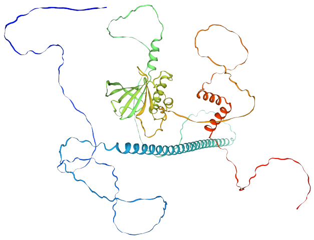

VaWRKY31-3

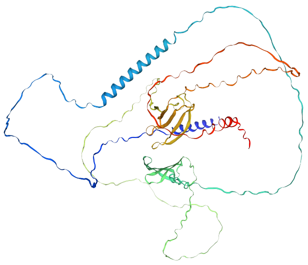

VaWRKY32

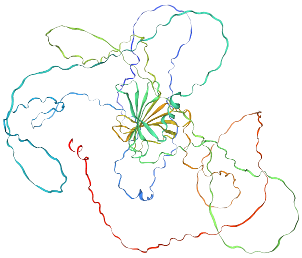

VaWRKY33-1

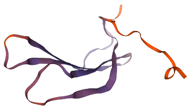

VaWRKY33-2

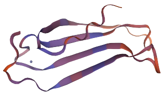

VaWRKY40-1

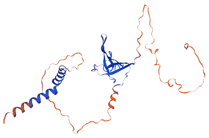

VaWRKY40-2

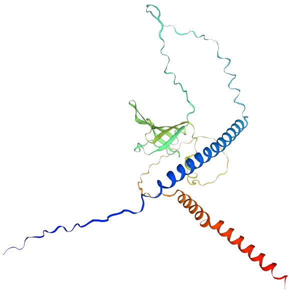

VaWRKY40-3

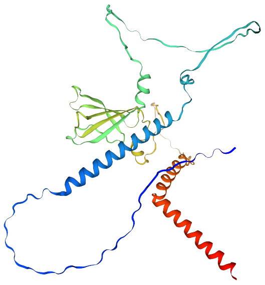

VaWRKY40-4

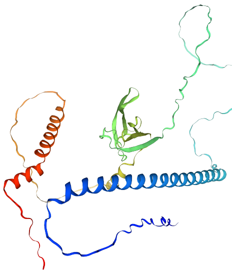

VaWRKY40-5

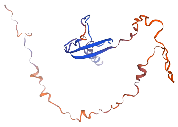

VaWRKY43-1

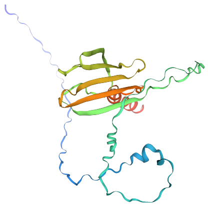

VaWRKY43-2

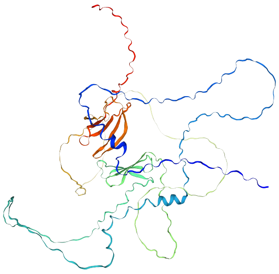

VaWRKY44-1

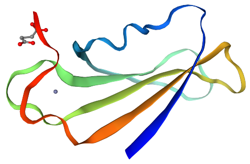

VaWRKY44-2

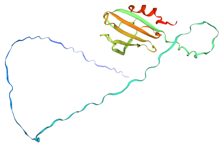

VaWRKY45

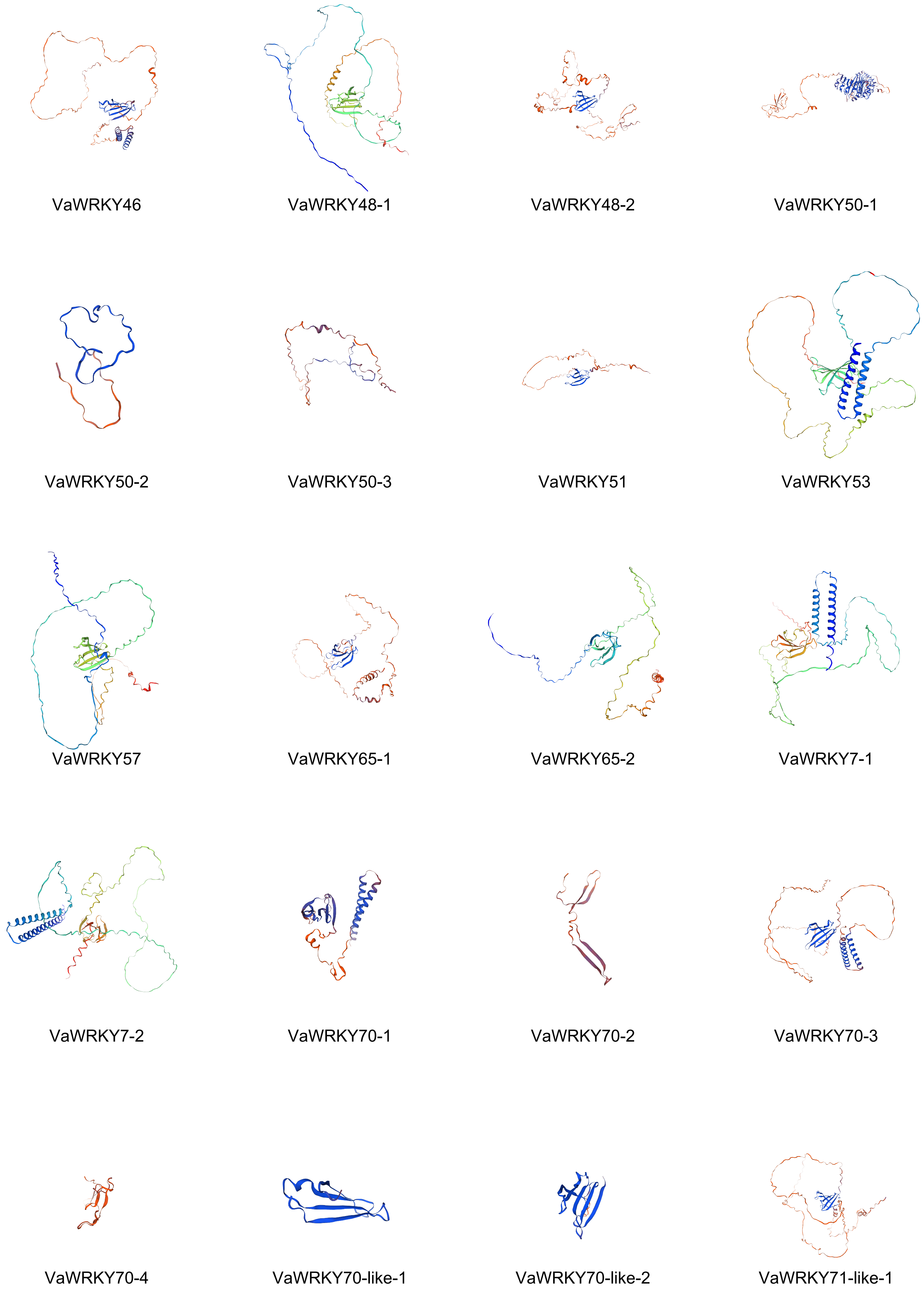
VaWRKY46
VaWRKY48-1
VaWRKY48-2
VaWRKY50-1
VaWRKY50-2
VaWRKY50-3
VaWRKY51
VaWRKY53
VaWRKY57
VaWRKY65-1
VaWRKY65-2
VaWRKY7-1
VaWRKY7-2
VaWRKY70-1
VaWRKY70-2
VaWRKY70-3
VaWRKY70-4
VaWRKY70-like-1
VaWRKY70-like-2
VaWRKY71-like-1

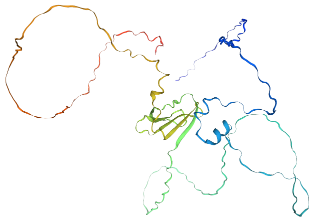

VaWRKY71-like-2

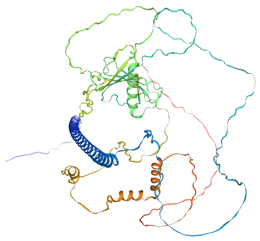

VaWRKY72A-like-1

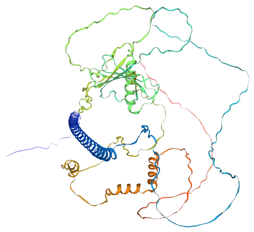

VaWRKY72A-like-2

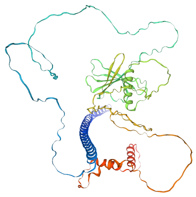

VaWRKY72A-like-3

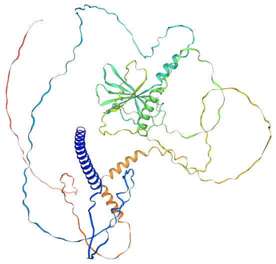

VaWRKY72B-like

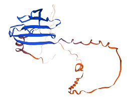

VaWRKY75

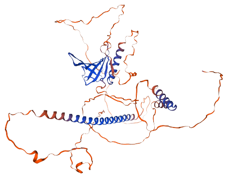

VaWRKY9

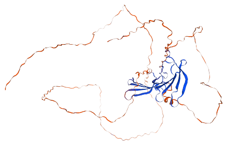

VaWRKYSUSIBA2
