# Supplementary material for: Genome-Wide Identification and Expression Analysis of the WRKY Gene Families in Vaccinium bracteatum
Source: Int J Mol Sci. 2025 Aug 13;26(16):7835. doi: 10.3390/ijms26167835 (PMC12386343; doi:10.3390/ijms26167835)
Supplement: Supplementary file 1 [file ijms-26-07835-s001.zip › Table S1 Identification of WRKY Families in V. bracteatum.pdf]

Table S1-1 Characterization of the *WRKY* Gene Families in *Vaccinium bracteatum* .

Name	Location	Aa	MW	pI	Asp + Glu
VaWRKY2-1	LG01:46929801..46936650-	727	78083.41	6.33	82
VaWRKY2-2	LG04:47571678..47576643-	686	74437.18	5.73	83
VaWRKY3-1	LG02:16488233..16495568-	535	57953.67	6.48	62
VaWRKY3-2	LG08:34971798..34976611+	522	57243.78	6.72	58
VaWRKY7-1	LG03:4885192..4888062-	325	35734.06	9.40	29
VaWRKY7-2	LG09:8036152..8038420-	347	38005.04	9.70	31
VaWRKY9	LG02:7803007..7805751-	522	57176.02	5.08	73
VaWRKY12	LG07:5706795..5713839+	235	26060.55	7.13	28
VaWRKY14	LG06:26503932..26507447+	482	52698.13	5.60	62
VaWRKY15	LG08:29955228..29958592-	324	35170.75	9.52	28
VaWRKY20-1	LG01:45748584..45753927+	593	64339.87	6.24	66
VaWRKY20-2	LG04:46276443..46283806+	567	62088.93	6.77	62
VaWRKY21-1	LG06:35963421..35966524+	353	39733.38	9.67	30
VaWRKY21-2	LG07:39068615..39070872-	265	29309.54	8.83	27
VaWRKY21-3	LG07:39073322..39076374-	339	38112.37	9.77	28
VaWRKY22-1	LG07:1100747..1102913+	316	35022.90	5.33	43
VaWRKY22-2	LG07:1764102..1766395-	322	35765.67	5.51	43
VaWRKY22-3	LG09:28986629..28989534+	350	38251.09	5.37	45
VaWRKY22-4	LG10:31083612..31085451+	348	37886.80	5.20	44
VaWRKY22-like-1	LG06:42758308..42760293+	273	31105.80	4.54	56
VaWRKY22-like-2	LG07:14412532..14413973-	341	37299.39	5.75	41
VaWRKY23	LG06:42886807..42890073+	376	41759.88	8.20	38
VaWRKY26	LG07:34962871..34966477-	542	59974.22	8.44	53
VaWRKY27	LG01:34620298..34620912+	204	23045.78	6.60	26
VaWRKY30	LG08:13613743..13614740+	192	22100.15	9.33	26
VaWRKY31-1	LG01:35902791..35905147-	491	53624.19	8.40	46
VaWRKY31-2	LG04:38314098..38319118-	537	58572.02	6.83	53
VaWRKY31-3	LG06:23330430..23333687-	572	62160.26	6.64	54
VaWRKY32	LG01:3363236..3373698-	516	57003.02	5.29	84
VaWRKY33-1	LG06:35704881..35720304-	820	90316.86	9.21	77
VaWRKY33-2	LG12:1396389..1401076+	552	61478.25	6.62	57
VaWRKY40-1	Contig00366:9861..16044-	233	26738.32	8.88	25
VaWRKY40-2	LG05:40580595..40583730-	245	27171.57	9.83	22
VaWRKY40-3	LG11:7608121..7611812-	287	32100.01	6.33	35
VaWRKY40-4	LG11:7693764..7696475+	341	38181.76	5.78	48
VaWRKY40-5	LG12:31994934..31997902-	328	36396.40	7.05	42
VaWRKY43-1	LG07:1466566..1468194+	182	20795.47	8.52	18
VaWRKY43-2	LG07:14380502..14382650-	198	22406.51	9.30	17
VaWRKY44-1	LG04:17504617..17511747-	474	51338.27	8.84	51
VaWRKY44-2	LG05:35353392..35357549+	189	21650.17	4.97	30

VaWRKY45	LG03:47776136..47778992-	172	19830.46	9.57	18
VaWRKY46	LG07:891195..893425+	320	35985.14	4.89	48
VaWRKY48-1	LG04:1557109..1559519-	342	38373.61	5.96	41
VaWRKY48-2	LG05:562157..564438-	356	39686.06	5.29	47
VaWRKY50-1	LG02:9352297..9354098-	590	67434.23	4.89	94
VaWRKY50-2	LG02:9358127..9358513-	75	8564.78	10.14	8
VaWRKY50-3	LG02:9365517..9367815-	145	16613.38	6.65	22
VaWRKY51	LG01:11845808..11850640-	192	21653.72	5.88	26
VaWRKY53	LG07:13755064..13757131+	450	51075.39	5.61	48
VaWRKY57	LG05:32520166..32531469+	314	34913.26	5.66	44
VaWRKY65-1	LG05:20692814..20696832+	287	31962.62	5.44	44
VaWRKY65-2	LG06:15319885..15321923-	268	29821.84	5.60	39
VaWRKY70-1	LG08:3374821..3378615-	185	21014.84	9.32	19
VaWRKY70-2	LG12:448933..451381+	331	36965.00	5.99	40
VaWRKY70-3	LG12:470216..472577+	333	37420.21	5.63	43
VaWRKY70-4	LG04:43705001..43710414+	297	32831.76	4.82	48
VaWRKY70-like-1	LG08:3293551..3298880+	344	38450.49	6.45	37
VaWRKY70-like-2	LG08:3347936..3349916+	320	35459.14	8.62	29
VaWRKY71-like-1	LG02:22386015..22389357-	333	37359.71	6.33	37
VaWRKY71-like-2	LG06:13823449..13825107-	317	35189.94	6.67	36
VaWRKY72A-like-1	LG08:13455223..13458516+	560	61138.01	7.59	57
VaWRKY72A-like-2	LG08:13477841..13481879+	559	60543.37	6.81	54
VaWRKY72A-like-3	LG08:13636336..13645921+	444	48575.10	8.36	45
VaWRKY72B-like	LG03:43427963..43434277-	567	61878.42	8.29	57
VaWRKY75	LG05:24751449..24754586+	187	21523.03	9.48	17
VaWRKYSUSIBA2	LG01:38268778..38273383+	481	52544.55	6.75	62

Table S1-2 Characterization of the *WRKY* Gene Families in *Vaccinium bracteatum* .

Name	Arg + Lys	Instability index	Aliphatic index	GRAVY	subcellular localization
VaWRKY2-1	75	50.53	60.52	−0.722	Nucleus
VaWRKY2-2	66	59.19	51.37	−0.802	Nucleus
VaWRKY3-1	58	58.61	54.92	−0.787	Nucleus
VaWRKY3-2	56	56.21	59.96	−0.744	Nucleus
VaWRKY7-1	43	35.73	58.83	−0.773	Nucleus
VaWRKY7-2	49	44.43	62.39	−0.627	Nucleus
VaWRKY9	54	49.57	59.85	−0.834	Nucleus
VaWRKY12	28	52.59	43.53	−0.959	Nucleus
VaWRKY14	49	53.65	53.03	−0.83	Nucleus
VaWRKY15	43	49.80	67.44	−0.53	Nucleus
VaWRKY20-1	58	53.85	62.93	−0.72	Nucleus
VaWRKY20-2	60	50.22	54.32	−0.746	Nucleus
VaWRKY21-1	51	49.54	66.54	−0.744	Nucleus
VaWRKY21-2	31	43.75	53.66	−0.863	Nucleus
VaWRKY21-3	48	55.11	68.44	−0.763	Nucleus
VaWRKY22-1	32	58.34	53.07	−0.798	Nucleus
VaWRKY22-2	33	61.52	49.94	−0.83	Nucleus
VaWRKY22-3	36	67.88	50.43	−0.81	Nucleus
VaWRKY22-4	34	63.01	60.55	−0.753	Nucleus
VaWRKY22-like-1	30	75.32	47.84	−1.184	Nucleus
VaWRKY22-like-2	34	58.01	65.51	−0.522	Nucleus
VaWRKY23	40	61.51	64.04	−0.661	Nucleus
VaWRKY26	56	55.04	41.94	−1.025	Nucleus
VaWRKY27	25	64.70	58.24	−0.842	Nucleus
VaWRKY30	35	38.65	62.97	−1.098	Nucleus
VaWRKY31-1	49	44.04	61.91	−0.652	Nucleus
VaWRKY31-2	50	45.67	65.47	−0.701	Nucleus
VaWRKY31-3	51	50.29	60.94	−0.639	Nucleus
VaWRKY32	65	57.30	61.43	−0.88	Nucleus
VaWRKY33-1	91	61.30	52.40	−0.83	Nucleus/ Chloroplast
VaWRKY33-2	55	61.28	41.34	−0.96	Nucleus
VaWRKY40-1	30	53.39	66.52	−0.694	Nucleus
VaWRKY40-2	38	47.82	58.57	−0.867	Nucleus
VaWRKY40-3	33	41.43	73.03	−0.717	Nucleus
VaWRKY40-4	41	52.51	71.17	−0.728	Nucleus
VaWRKY40-5	42	47.40	64.51	−0.821	Nucleus
VaWRKY43-1	20	52.79	61.59	−0.829	Nucleus
VaWRKY43-2	24	40.47	64.44	−0.699	Nucleus
VaWRKY44-1	60	46.14	62.32	−0.806	Nucleus
VaWRKY44-2	19	39.62	64.97	−0.635	Nucleus

VaWRKY45	29	39.97	50.41	−0.926	Nucleus
VaWRKY46	32	38.55	66.41	−0.599	Nucleus
VaWRKY48-1	35	63.72	45.56	−0.931	Nucleus
VaWRKY48-2	36	61.64	52.33	−0.756	Nucleus
VaWRKY50-1	62	47.83	99.14	−0.223	Nucleus/Cell membrane
VaWRKY50-2	17	42.05	49.47	−0.92	Nucleus
VaWRKY50-3	21	54.50	46.48	−1.046	Nucleus
VaWRKY51	21	58.63	45.62	−0.951	Nucleus
VaWRKY53	31	52.11	56.13	−0.711	Nucleus
VaWRKY57	36	68.51	44.75	−0.958	Nucleus
VaWRKY65-1	37	62.52	59.72	−0.79	Nucleus
VaWRKY65-2	32	64.43	52.69	−0.824	Nucleus
VaWRKY70-1	26	47.45	70.59	−0.68	Nucleus
VaWRKY70-2	32	61.41	65.08	−0.721	Nucleus
VaWRKY70-3	33	56.06	60.00	−0.825	Nucleus
VaWRKY70-4	30	47.95	49.56	−0.871	Nucleus
VaWRKY70-like-1	34	64.75	51.28	−0.726	Nucleus
VaWRKY70-like-2	32	59.77	53.59	−0.826	Nucleus
VaWRKY71-like-1	31	69.76	42.49	−1.052	Nucleus
VaWRKY71-like-2	35	67.24	48.01	−0.863	Nucleus
VaWRKY72A-like-1	58	51.17	62.59	−0.667	Nucleus
VaWRKY72A-like-2	53	44.59	61.82	−0.643	Nucleus
VaWRKY72A-like-3	48	37.65	67.68	−0.689	Nucleus
VaWRKY72B-like	60	50.18	60.95	−0.771	Nucleus
VaWRKY75	27	39.60	47.38	−1.034	Nucleus
VaWRKYSUSIBA2	60	57.65	57.73	−1.012	Nucleus
