# Supplementary material for: Genome-Wide Identification and Expression Analysis of the WRKY Gene Families in Vaccinium bracteatum
Source: Int J Mol Sci. 2025 Aug 13;26(16):7835. doi: 10.3390/ijms26167835 (PMC12386343; doi:10.3390/ijms26167835)
Supplement: Supplementary file 1 [file ijms-26-07835-s001.zip › Table S3 Gene ID and Name.pdf]

Table S3 Gene ID and Name

| Gene ID | Name | Gene ID | Name |
|---|---|---|---|
| EVM0001409.1 | *VaWRKY50-2* | EVM0002398.1 | *VaWRKY12* |
| EVM0028918.1 | *VaWRKY50-1* | EVM0026882.2 | *VaWRKY65-2* |
| EVM0013640.1 | *VaWRKY50-3* | EVM0027914.1 | *VaWRKY71-like-2* |
| EVM0004908.3 | *VaWRKY2-2* | EVM0004835.1 | *VaWRKY31-3* |
| EVM0020314.1 | *VaWRKY20-2* | EVM0014407.1 | *VaWRKY26* |
| EVM0011379.1 | *VaWRKY2-1* | EVM0035071.1 | *VaWRKY44-2* |
| EVM0008439.1 | *VaWRKY45* | EVM0032263.1 | *VaWRKY21-3* |
| EVM0025614.1 | *VaWRKY46* | EVM0002334.1 | *VaWRKY21-2* |
| EVM0018028.1 | *VaWRKY22-1* | EVM0014456.1 | *VaWRKY40-5* |
| EVM0009951.1 | *VaWRKY43-1* | EVM0002115.1 | *VaWRKY48-1* |
| EVM0034397.1 | *VaWRKY22-3* | EVM0015342.1 | *VaWRKY7-1* |
| EVM0011958.1 | *VaWRKY70-1* | EVM0031657.1 | *VaWRKY3-2* |
| EVM0021199.1 | *VaWRKY70-like-2* | EVM0002092.1 | *VaWRKY53* |
| EVM0031566.1 | *VaWRKY70-like-1* | EVM0003016.1 | *VaWRKY43-2* |
| EVM0032647.1 | *VaWRKY75* | EVM0011639.1 | *VaWRKY22-like-2* |
| EVM0005542.1 | *VaWRKY71-like-1* | EVM0022578.1 | *VaWRKY33-1* |
| EVM0009926.1 | *VaWRKY40-2* | EVM0025889.1 | *VaWRKY21-1* |
| EVM0027469.1 | *VaWRKY33-2* | EVM0020899.1 | *VaWRKY31-1* |
| EVM0024717.1 | *VaWRKY70-2* | EVM0022818.1 | *VaWRKY57* |
| EVM0006080.1 | *VaWRKY70-3* | EVM0028944.1 | *VaWRKY27* |
| EVM0000749.1 | *VaWRKY65-1* | EVM0023988.1 | *VaWRKY7-2* |
| EVM0004522.1 | *VaWRKY23* | EVM0019263.2 | *VaWRKY40-4* |
| EVM0015471.1 | *VaWRKY22-like-1* | EVM0011228.1 | *VaWRKY40-3* |
| EVM0035549.1 | *VaWRKYSUSIBA2* | EVM0013542.1 | *VaWRKY22-2* |
| EVM0012286.1 | *VaWRKY14* | EVM0003030.1 | *VaWRKY72A-like-3* |
| EVM0002852.1 | *VaWRKY31-2* | EVM0005280.1 | *VaWRKY72A-like-2* |
| EVM0011208.1 | *VaWRKY20-1* | EVM0014100.1 | *VaWRKY72A-like-1* |
| EVM0007637.1 | *VaWRKY70-4* | EVM0028691.1 | *VaWRKY30* |
| EVM0024191.1 | *VaWRKY40-1* | EVM0001089.1 | *VaWRKY72B-like* |
| EVM0017037.2 | *VaWRKY9* | EVM0035111.1 | *VaWRKY32* |
| EVM0026975.1 | *VaWRKY22-4* | EVM0026792.1 | *VaWRKY44-1* |
| EVM0016305.1 | *VaWRKY51* | EVM0002118.1 | *VaWRKY15* |
| EVM0007099.1 | *VaWRKY48-2* | EVM0004259.1 | *VaWRKY3-1* |
