# Supplementary material for: Genome-Wide Identification and Expression Analysis of the WRKY Gene Families in Vaccinium bracteatum
Source: Int J Mol Sci. 2025 Aug 13;26(16):7835. doi: 10.3390/ijms26167835 (PMC12386343; doi:10.3390/ijms26167835)
Supplement: Supplementary file 1 [file ijms-26-07835-s001.zip › TABLES~2.pdf]

Table S2 The *VaWRKY* gene significantly differentially expressed during different stages of *V.bracteatum* fruit ripening.

Function	NAME	Organism
Upregulated at each stage	VaWRKY23	*Camellia sinensis*
	VaWRKY32	*Rhododendron vialii*
	VaWRKY45	*Rhododendron vialii*
	VaWRKY20-1	*Rhododendron vialii*
	VaWRKY22-2	*Rhododendron vialii*
Upregulated in the blue-fruit stage	VaWRKY44-2	*Magnolia sinica*
	VaWRKY72A-like-3	*Rhododendron vialii*
Increase after a decrease	VaWRKY57	*Rhododendron vialii*
	VaWRKY21-1	*Actinidia eriantha*
Reduced at each phase	VaWRKY65-1	*Camellia sinensis*
	VaWRKY3-2	*Rhododendron vialii*
	VaWRKY22-1	*Rhododendron vialii*
