# Supplementary figures and images for: Genome-Wide Identification and Expression Analysis of the WRKY Gene Families in Vaccinium bracteatum
Source: Int J Mol Sci. 2025 Aug 13;26(16):7835. doi: 10.3390/ijms26167835 (PMC12386343; doi:10.3390/ijms26167835)

***EVM0002988***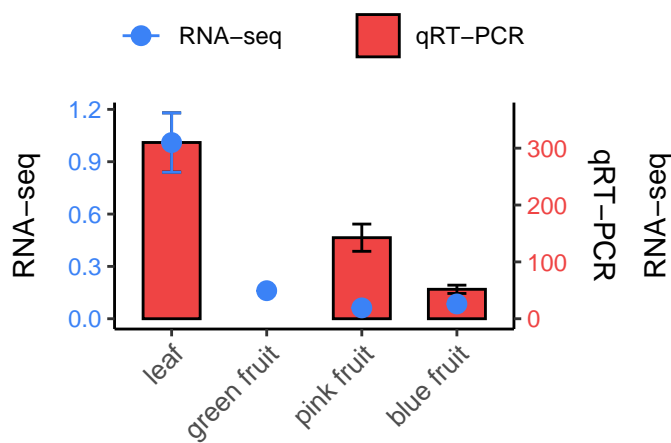***EVM0004767***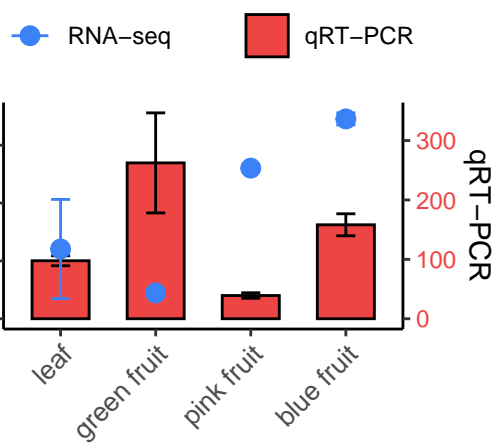***EVM0013373***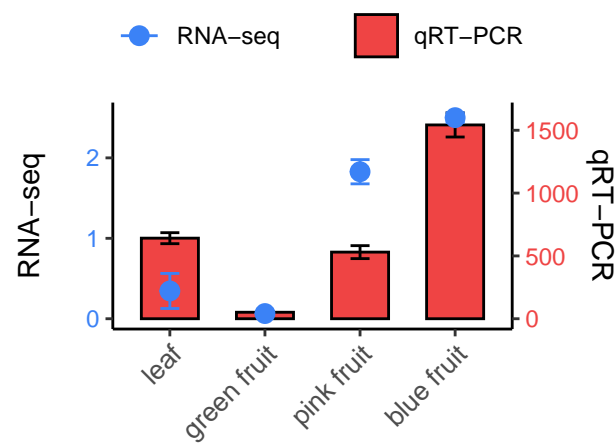***EVM0013821***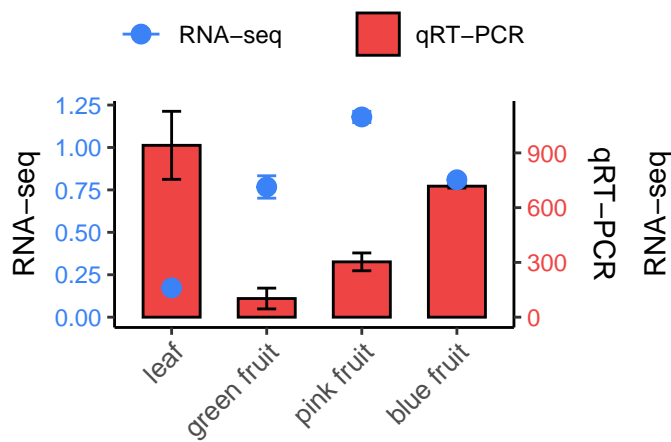***EVM0015464***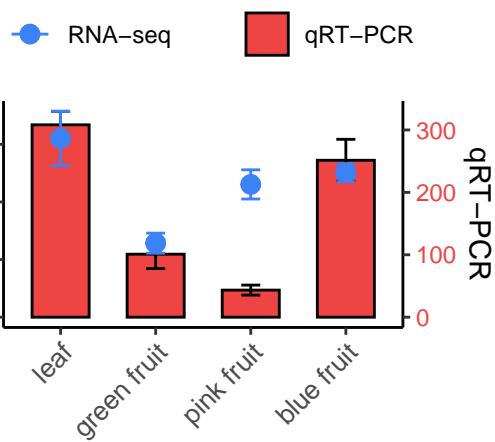***EVM0025897***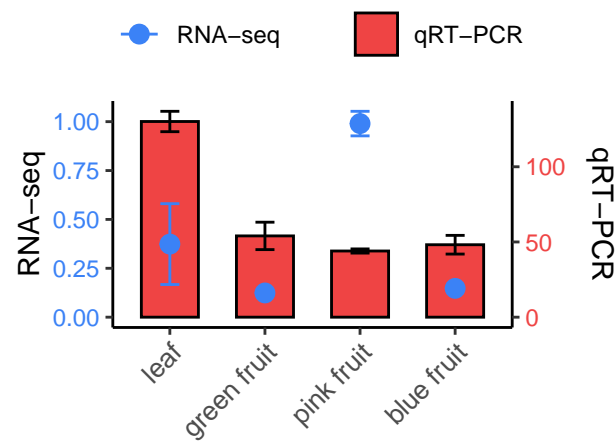***EVM0026502***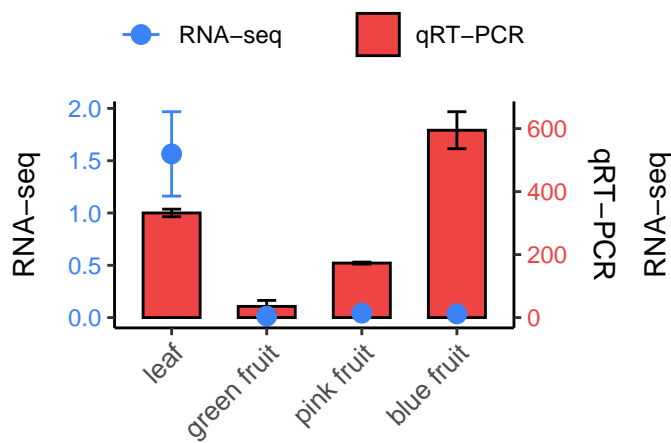***EVM0004099***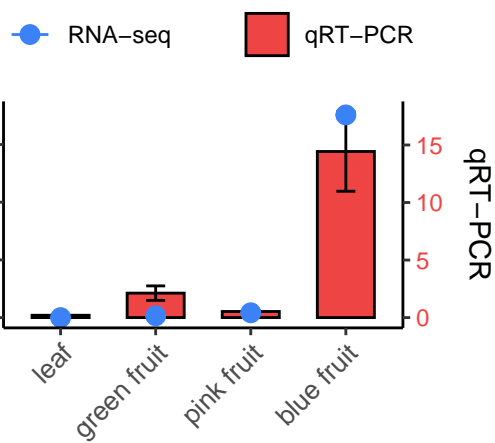***EVM0015476***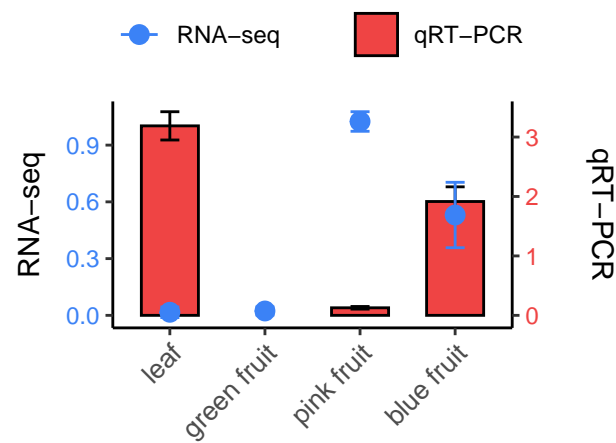***EVM0015559***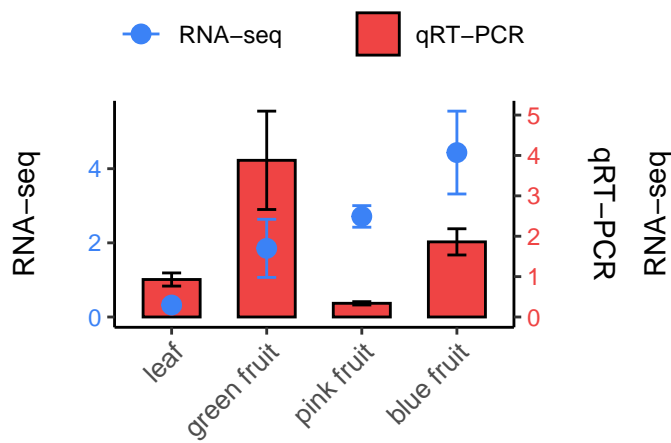***EVM0021601***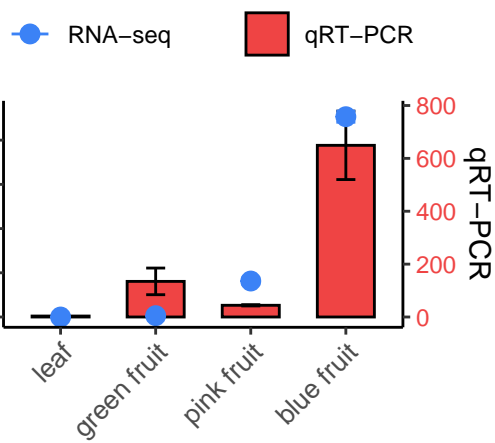***EVM0024658***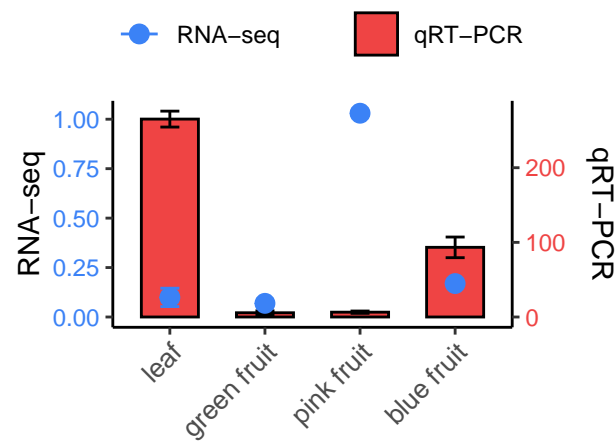***EVM0027149***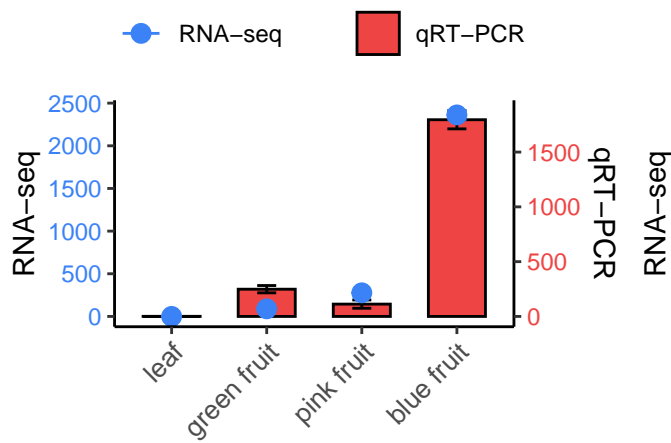***EVM0035149***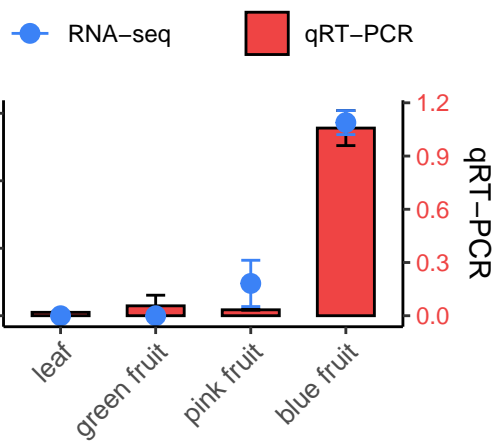

Supplement: Supplementary file 1 [file ijms-26-07835-s001.zip › arious tissues of Vaccinium brteatum.pdf]
